# Accumulated effects of factors determining plant development from somatic embryos of *Abies nordmanniana* and *Abies bornmuelleriana*

**DOI:** 10.3389/fpls.2022.989484

**Published:** 2022-10-13

**Authors:** Ulrik Braüner Nielsen, Camilla Bülow Hansen, Ulrich Hansen, Vivian Kvist Johansen, Ulrika Egertsdotter

**Affiliations:** ^1^ Department of Geosciences and Natural Resource Management, University of Copenhagen, Frederiksberg, Denmark; ^2^ Hansen Skovplant, Frankfri, Denmark; ^3^ Department of Forest Genetics and Plant Physiology, Umeå Plant Science Centre, Swedish University of Agricultural Sciences, Umeå, Sweden; ^4^ Renewable Bioproducts Institute, Georgia Institute of Technology, Atlanta, GA, United States

**Keywords:** *abies*, conifer, *in vitro*, germination, embryo quality, nordmann fir, turkish fir

## Abstract

Despite a much later inception of somatic embryogenesis (SE) propagation protocols for gymnosperms than for angiosperm species, SE is becoming increasingly important due to its applications for commercial forestry. For many conifers, there are however still major bottlenecks in the SE plant production process limiting the use of SE for forestry operations, Christmas tree production and research projects. In the present case study, the effects on plant growth from different cultural factors applied during the SE developmental process were studied in two conifer species of high value for Christmas tree production. Seven clones of *Abies nordmanniana* and two clones of *Abies bornmuelleriana* were included in the study. Accumulated effects from cultural treatments were recorded from the start of germination of mature embryos of different quality scores through development into plants in the third growing period. Experimental factors of the cultural treatments included were: germination temperature, germination time, light conditions, survival ex vitro and traits for plant growth and vitality. The results reveal that most of the studied experimental factors influenced plant growth during the first three years however their relative importance was different. Plant survival rate at end of the nursery stage was strongly impacted by germination temperature (p<0.001), initial embryo score (p=0.007), clone (p<0.001) and to a lesser extend week of germination (p=0.017). This case-study highlights and quantifies the strong interrelation between the developmental steps of somatic embryogenesis and show the importance of considering all cultural steps when optimizing SE plant production protocols.

## 1 Introduction

Successful somatic embryogenesis (SE) in conifer species was first demonstrated in the mid-80s in *Picea abies* (Chalupa, 1985; Hakman et al., 1985) and *Larix decidua* (Nagmani and Bonga, 1985). Protocols for conifer SE plant production have since then been expanded to other conifers still mostly within *Pinaceae*, but also some from *Cupressaceae, Taxaceae, Cephalotaxaceae*, and *Araucariaceae* families (Klimaszewska et al., 2016). The methods have also been gradually improved with the overall goal to meet the needs and expectations from forestry operations where SE can be successfully used as a clonal propagation method to support breeding programs (Dungey et al., 2009; Resende et al., 2014) and for propagation of elite trees (Park, 2002) or for clonal propagation of families through ‘family forestry’ (Rosvall et al., 1998; Lindgren, 2008).

Clonal propagation of conifers is also possible by cuttings but there are issues for scale up due to early ageing of the donor plants limiting the time for cutting production, plagiotropic growth and high labor cost (White et al., 2007; Rasmussen et al., 2019). The SE method has many advantages such as the possibility to store valuable germplasm for extended periods of time by cryopreservation (Bonga, 2015) and scale up production supported by automation (Egertsdotter et al., 2019). Furthermore, the possibility to apply genomic selection for traits to early stages of the SE process has a high potential for shortening breeding cycles and increase wood production as was recently demonstrated for *Picea glauca* (Chamberland et al., 2020). Genomic selection may also be possible for specific traits such as height growth in *Pinus taeda* L. (Lauer et al., 2022) and drought stress in *Sequoiadendron giganteum* and *Sequoia sempervirens* (De La Torre et al., 2022). The recent breakthrough in genomic modification by CRISPR/Cas9 in *Pinus radiata* (Poovaiah et al., 2021) and *Picea galuca* (Ying et al., 2021) further increases the potential of the SE system for designing well-adapted trees for future forests. In research studies, SE provides an important tool for generating clonal plants for field trials of conifer species where clonal propagation by cuttings otherwise is limited (Rasmussen et al., 2019). SE is also used in fundamental studies on embryo development of the much less studied gymnosperm embryogenesis that has been shown to differ from angiosperm embryogenesis in many important respects (Uddenberg et al., 2015).

Another area of application for conifer SE is for Christmas tree production, which overwhelmingly involves conifer species and where the most favoured tree species come from within the genus *Abies*. Christmas trees are grown as short-rotation crops typically for five to ten years depending on species and geographical location which makes them a superior study system for conifer SE protocol improvements and evaluation of clonal selections relative to other conifers with much longer rotation times of at least 15 years in the Southern hemisphere (*Pinus radiata*) and on average 60 years in northern Europe and Canada (*Picea*–species). In Europe, *Abies nordmanniana* (Steven) Spach is the most commonly used Christmas tree with an estimated yearly demand of 35-40 million trees (Christensen, 2016). The closely related species *Abies bornmuelleriana* (Mattf.) (Liu, 1971) has gained increasing interest in Denmark due to a new superior-performing seed source (Nielsen, 2021) and less sensitivity to the adelgid *Dreyfusa nordmanninana* (Nielsen et al., 2017). These two commercially valuable *Abies*-species were selected for the present study aimed at dissecting the relative importance of different factors for SE development, starting with the morphology of the mature embryo up to the fully developed, growing tree, with the focus on temperature-effects during germination.

The SE process in conifers requires several consecutive cultural stages starting with the initiation of the earliest-stage of somatic embryos in culture (proembryogenic masses; PEMs) from the initial explants (zygotic embryos); multiplication of the PEMs (resulting in proliferation of the ‘SE culture’), maturation of PEMs followed by germination of mature embryos and ex vitro growth of planted germinants (Egertsdotter, 2019). There are however still several major bottlenecks in the SE process limiting cost-effective use of SE for producing plants for forestry, Christmas tree production and research projects. Recent efforts have been made to achieve cost efficiency by scale up supported by automation and alternative culture techniques (Egertsdotter et al., 2019, Valdiani et al., 2019).

The first bottleneck of the SE-process is the initiation step when PEMs are induced to form from the zygotic embryo extracted from a seed. Several studies have shown that the genetic background of the mother tree affects the success rate of SE initiation. Relatively large variances due to specific combining ability and maternal effects were found for the initiation of SE for *Picea glauca* (Park et al., 1993; Park et al., 1994) and *Pinus taeda* (MacKay et al., 2006). Large genetic variation for SE initiation among families was also observed in *Picea mariana* (Cheliak and Klimaszewska, 1991). In open-pollinated trees of *Pinus radiata*, the same top three mother trees out of seven tested were shown to maintain their SE initiation potential over the two years tested (Montalbán et al., 2012). In *Abies nordmanniana*, only 2-15.5% of the seeds from two mother trees could be induced to form somatic embryos demonstrating large differences with respect to potential for SE propagation (Nawrot-Chorabik, 2016) whereas in *A. bornmuelleriana*, the SE initiation rate was on average 25-35% from 1300 mature seeds derived from six mother trees where all trees gave initiations (Nielsen et al., 2018).

The second step which is the proliferation phase when the early-stage plant propagules (PEMs) multiply, is in most cases not a limiting step once the culture of PEMs has been established from the first initiation and started to proliferate.

For the third developmental stage of embryo maturation, large differences between species and clones within species are seen. The variability in maturation yields is at least partially accounted for by the lack of synchronization during the maturation process allowing only a fraction of the developing embryos present in the culture to form mature embryos that can germinate (Mamun et al., 2018).

There are relatively fewer studies that have focused on the factors affecting the consecutive steps of germination of mature embryos and establishment of plants growing *ex vitro*. Efforts to establish image analysis-based tools to correlate the morphology of the mature embryo to germination success have been done in connection with scale up and automation efforts (reviewed in Egertsdotter et al., 2019, Le et al., 2021).

Improvements of the conifer SE process have overwhelmingly been focused on the composition of culture media at different stages of development (Klimaszewska et al., 2016). Different growing conditions with respect to light (Nawrot-Chorabik, 2016; Varis et al., 2021) and desiccation treatments (Krajňáková and Häggman, 2016) have also been explored.

The effect of germination temperature on somatic embryo germination success and vigor of the subsequent plants was demonstrated in *Picea abies* where germination success was improved by cold storage (Tikkinen et al., 2018b). Germination rates were also improved by a cold treatment at 4°C for *A. fraseri* (Pullman et al., 2016) and by a cold treatment for three weeks during desiccation at 4°C in *A. cephalonica* (Krajňáková and Häggman, 2016).

It has been previously shown that the temperature during embryo development affects the time of bud burst and bud set both during zygotic (Johnsen et al., 2005) and somatic embryogenesis (Kvaalen and Johnsen, 2008). Subsequent analyses of the different temperature-induced epitypes showed differential expression of genes related to DNA and histone methylation, the sRNA pathway and putative thermos sensing genes thus suggesting that the temperature-effects were epigenetically regulated (Yakovlev et al., 2011). A similar effect from temperature during embryo development was observed for bud burst in somatic seedlings from *Abies nordmanniana* (Lobo et al., 2022).

Each step of the SE process has an impact on the yield and quality of the final plant in the ground: the types of the PEMs present in the SE culture affect the yields of mature embryos; the morphology of the mature embryos is correlated to the success of germination and root and shoot growth, and vitality of the germinant is essential for acclimatization and survival ex vitro (Egertsdotter, 2019). Numerous studies have been dedicated to improving the different steps of the SE process but to our knowledge, to date none has considered the accumulated effects from several steps on plant growth over several growing seasons.

The objectives of our study were to analyze and quantify the effect of clonal identity, embryo quality, germination temperature and time for germination, light, germinant quality and specifically the accumulated effect of these factors on the vitality of plants from the first to the third growing season. New approaches to optimize SE plant production of the two targeted *Abies* species based on the results from this study are discussed.

## 2 Materials and methods

### 2.1 Plant material

Embryogenic cell lines representing seven clones of *Abies nordmanniana* and two clones of *Abies bornmuelleriana* were included in the study. The embryogenic cultures of *A. nordmanniana* originate from seeds harvested at different locations: 1) seeds directly imported in 2010 from Caucasus, Georgia, Ambrolauri Tlugi (family 7, line N1) and 2) seeds harvested in 2003 from a Danish approved seed stand F.527 Tversted (family 15, lines N3, N6 and family 16, lines N2, N4, N5 and N7). 3) For *A. bornmuelleriana*, seeds were harvested in 2016 from a commercial Danish approved seed orchard FP.267 Kongsøre (lines T1 and T2). A total of 3097 embryos were included in the study (**Table 1**).

**Table 1:** Overview of clones, number of embryos and treatments at the start of germination at different temperatures.

Species	clone	temperature °C							
		2	4	5	8	10	15	20	Total
***Abies nordmanniana***									
	**N1**	41	60	60	40	40	40	40	321
	**N2**	40	80	80	40	40	40	39	359
	**N3**	40	80	80	40	40	40	40	360
	**N4**	40	60	60	40	40	40	40	320
	**N5**	40	59	60	40	40	40	40	319
	**N6**	40	60	60	40	40	40	40	320
	**N7**	40	40	40	40	40	40	40	280
***Abies bornmuelleriana***									
	**T1**	40	140	120	40	40	40	40	460
	**T2**	40	79	79	40	40	40	40	358
		361	658	639	360	360	360	359	3097

Embryogenic cultures used in the present study were either thawed from cryogenic storage where *A. nordmanniana* cell lines in groups 1) and 2) had been cryo-stored in 2011 and 2003, respectively, and *A. bornmuelleriana* cell lines in group 3) were used for the experiments directly after initiation from fresh, immature seeds (Valdiani et al., 2019).

**Figure 1:**
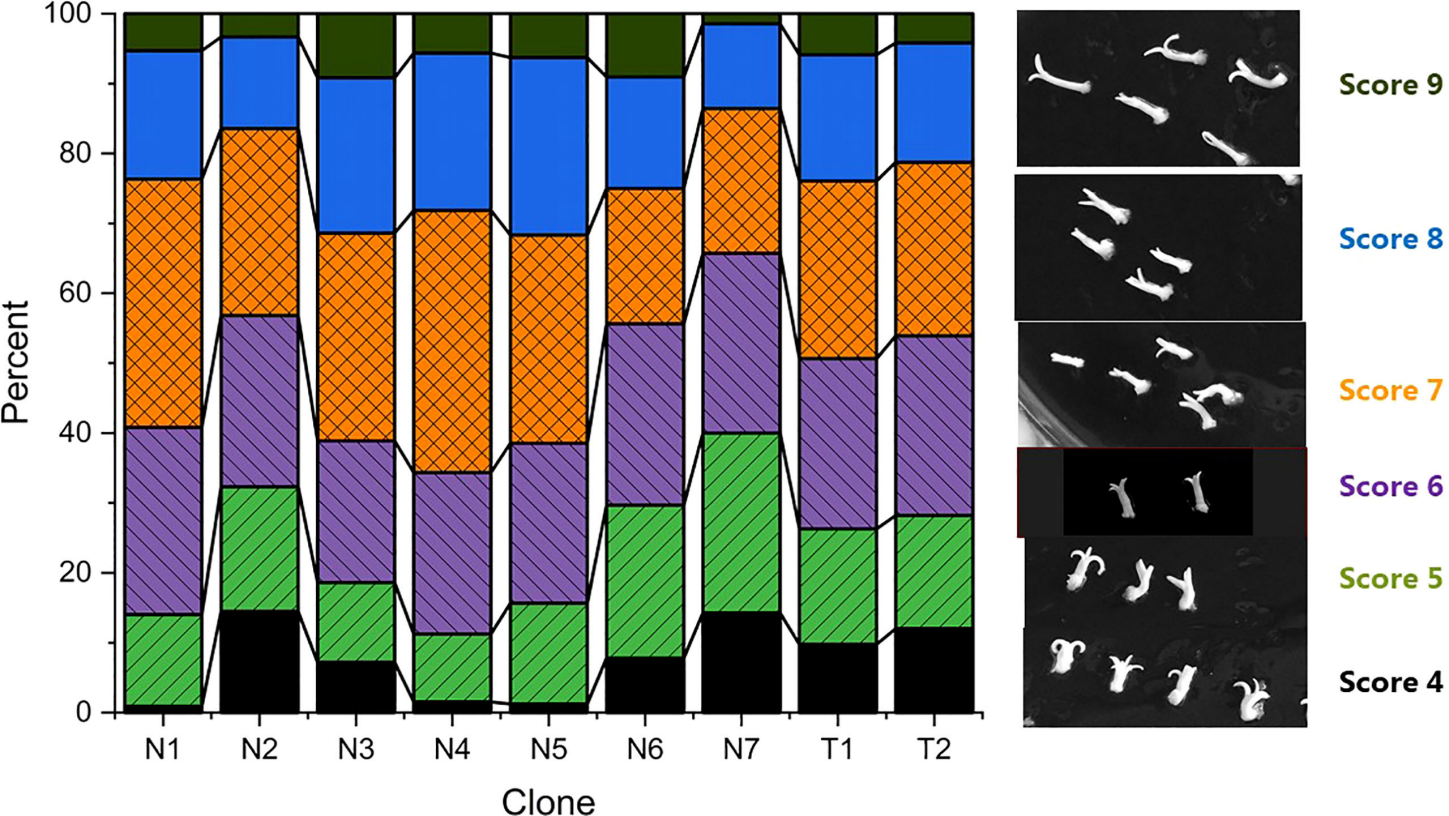
Quality scores for embryos at the start of germination (a total of 3097) and photos of typical embryo morphologies corresponding to each scoring category. Percentage of embryos of scores 4-9 within each clone.

Methods for initiation and proliferation were performed according to Valdiani et al. (2019) and maturation according to Find (2020). Three consecutive media were used for the maturation process: M1 (Medium 49.53), M2 (Medium 29.75) and M3 (Medium 8.95). Briefly, to start maturation, 4 g of proliferating embryogenic culture was dispersed in 100 ml of liquid proliferation medium by running a blender (Waring laboratory, Variable speed laboratory blender, LB20, velocity settings 500-22000 rpm) at speed position between mark 1 and 2 for 25 seconds. After 30 minutes of sedimentation, 70 ml of liquid were removed from the top and 1 ml of the remaining suspension containing approximately 40 mg of embryogenic culture was pipetted onto a filter paper (Th. Geyer, 70mm, Grade 54) in a Petri plate with M1. The filter papers with maturing embryo cultures were moved between maturation media in the order of M1-M2-M1-M3 after three, three, six and two weeks, respectively. The cultures were kept at 22°C in darkness during the 12-week maturation period. Before the start of the germination experiments, the mature embryos were scored into nine categories based on the morphologies of the cotyledons and hypocotyls (**Table 2** and **Figure 1**). Scores 1-3 were later omitted because they had very low or no germination response (data not shown). A total of 3097 mature embryos with scores 4 to 9 (photo panel showing each embryo after 0, 3 and 8 weeks, see supplementary material) were included in the study, and distribution of embryo scores within clones is shown in **Figure 1**.

**Table 2:** Initial embryo quality score recorded before starting germination treatment.

Category	Initial embryo score
**Good quality:** *Slim hypocotyl, minimum two cotyledons*	9 = Hypocotyl longer or equal to 6.0 mm
	8 = Hypocotyl 4.0-5.99 mm
	7 = Hypocotyl less than or equal to 3.99 mm
**Medium quality**: *Slightly swollen hypocotyl, minimum two cotyledons*	6 = Hypocotyl longer or equal to 4.0 mm
	5 = Hypocotyl 2.0-3.99 mm
	4 = Hypocotyl less than or equal to 1.99 mm
**Poor quality:** *Embryos deformed*	3 = Looks like an embryo, but too many cotyledons, swollen hypocotyl, small and bent
	2 = Only long hypocotyl, either straight, bent or shrimp-like
	1 = Deformed embryos, cotyledons grow from center of hypocotyl, cotyledons deformed

### 2.2 Germination of mature somatic embryos

**Figure 2:**
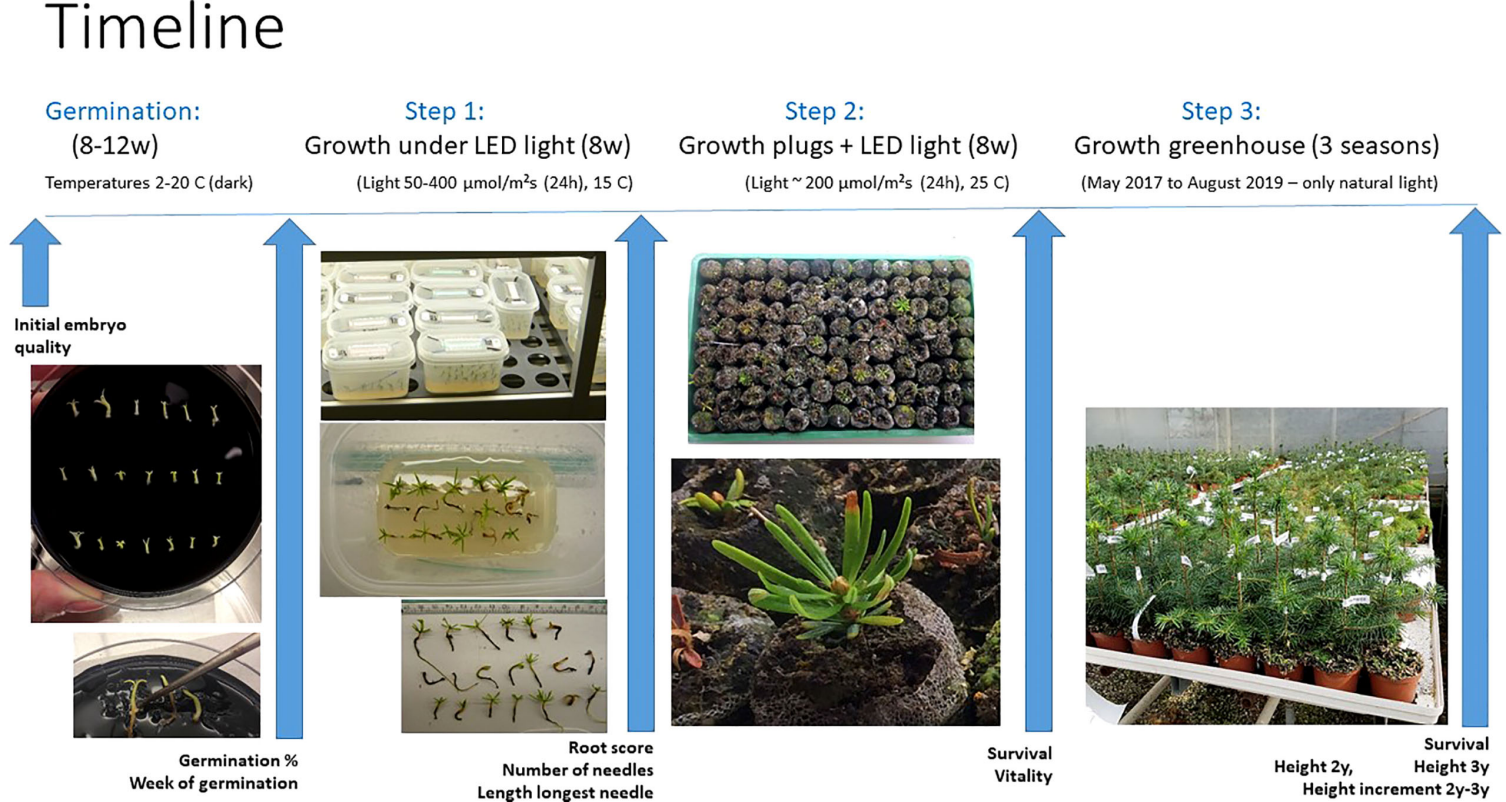
Overview of the timeline for the SE process steps monitored in the study. Germination of embryos for 8-12 weeks in darkness at different temperatures between 2°C to 20°C. Step 1: plant growth for 8 weeks under LED light (50-400 µmol m^-2^s-^1^¸24 h) at 15°C. Step 2: plant growth after transplanting into peat plugs for 8 weeks under LED light (180-200 µmol m^-2^ s^-1^, 24 h) at 25°C. Step 3: plant growth for 3 years in a commercial nursery with natural light. The measurements carried out at each step are defined under the blue arrows.

Medium 51.21 (Find, 2020) was used for germination. In each Petri dish, six embryos were placed in each of three rows (**Figure 2**, Germination) with embryos: 1-6 in the top row, 7-13 in the second row and 14-20 in the third row. The initial numbering of embryos in the Petri plates was maintained throughout the experiment such that at the end of the experiment, plants could be traced to individual embryo identification numbers and subsequent treatments. Embryos were placed horizontally on the surface of the germination medium and germinated for 8-12 weeks in darkness at temperatures 2, 4, 5, 8, 10, 15 and 20°C. The number of embryos per clone and treatment varied (**Table 1**). For the temperature treatments of 2 to 10°C, five standard refrigerators (Wasco, model 16973, K110W) with a temperature sensor overruling the internal refrigerator temperature sensor (Renkforce, UT300 Universal-Thermostat -40 to 99°C) were used securing stable temperatures within +/- 0.2°C. Additionally, a fan was added to distribute the air and prevent layering of the temperature within the refrigerator. The treatments at 15°C and 20°C were performed in a refrigerated growth chamber (range +/- 2°C), and a basement at room temperature (+/-2°C), respectively. Temperatures were logged continuously (Hobo Onset Pendant ^®^ Temperature/light 64k datalogger, UA-002-64). Photos were taken from all Petri dishes at weeks 0, 3, 5, 8-12. Root growth was monitored weekly from week 8 to 12. When the root had developed 5 mm or more, germinated embryos were planted for photoautotrophic growth as described below.

### 2.3 Growth and development of planted germinated embryos

Acclimatization and subsequent growth ex vitro of a total of 1150 planted germinated embryos were tested in three sequential steps: 1) 8 weeks of photoautotrophic growth on sugar-free germination medium under sterile conditions with different LED light intensities (24h) at 15°C, 2) after transplanting plantlets into peat plugs (Jiffy7 Forest plugs, 25 mm), an additional 8 weeks under one intensity of LED light (24h) at 25°C, and 3) final transfer to a greenhouse for growth during three seasons (**Tables S1, S2**). Identity-records of each of the germinated embryos were kept respectively through all three steps of the experiment. An overview and timeline of the full experimental set up is shown in **Figure 2**.

#### 2.3.1 Photoautotrophic growth under different continuous light intensities (step 1)

During weeks 8 to 11 of germination, germinated embryos were moved to aerated boxes (Eco2box, oval, Duchefa Biochemie) in a way that germinated embryos from a specific batch (batches of 20 embryos per Petri dish having the same treatment and clone during germination) were separated and placed in separate boxes keeping embryo traceability. Each aerated box contained 100 ml of a semisolid sugar free medium (Find, 2020). Twenty germinated embryos were planted vertically into lines pre-cut with a knife and evenly spaced in three rows of respectively 6, 7 and 7 in each box.

The positions of the aerated boxes were randomized under seven light intensities: 50, 100, 150, 200, 250, 300 and 400 μmol m^-2^ s^-1^ of continuous light (24h) (LED lights, 1500*32*32mm, T8 LED Tube, Lumen 2500LM, CCT: 4000K, Ra 80+, 26W) with the same number of boxes under each light intensity at 15°C. Since the germination process lasted four weeks, the process above was repeated each week at the transfer of germinated embryos resulting in four batches, one for each week of harvest of germinated embryos (8, 9, 10 and 11). After 8 weeks under LED light, photos were taken of each box from above and of individual germinated embryos removed from the boxes (**Figure 2**, step 1). Quality of the somatic plant was assessed by length of the root tip, and the number and length of young needles. The root quality was evaluated based on the photos according to the following criteria: 0 = no white root tip; 1 = white root tip less than 2 mm; 2 = white root tip longer or equal to 2 mm; K = root tip broken and cannot be evaluated. The number of young needles, and length of the longest young needle were recorded using the program Klonk Image Measurement ^®^ (https://www.imagemeasurement.com, free download).

#### 2.3.2 Growth under continuous light (step 2)

All plants from the previous step of photoautotrophic growth were transplanted into Jiffy7 Forest plugs (25 mm) and placed in a tray (Mini Greenhouse, Biltema) holding 8x13 plugs. Each tray then contained plants from four boxes (and some empty plugs to keep growing conditions uniform; **Figure 2**). Before planting, plugs were soaked with demineralized water with added NPK fertilizer (Azelis Pioner Hvid 18-2-15 plus micronutrients, https://tanggard.dk/wp-content/uploads/2020/01/Pioner-Basis-Hvid.pdf) to a conductivity of 1.5 S m^-1^ and pH 4.5. Trays were placed under continuous (24h) LED light with an intensity range of 175-195 μmol m^-2^ s^-1^ at 25°C and watered (without fertilizer) three times a week to keep plug humidity. The trays were kept covered with a lid for six weeks, in week seven the lids were slightly raised and removed in week 8. Plant survival and vitality was recorded after 8 weeks. The surviving plants were scored into three categories: good and vigorous (score 9-8-7), acceptable (score 6-5-4) and poor (score 3-2) (**Table S1**).

#### 2.3.3 Growth in greenhouse without artificial light (step 3)

The plants in plugs were acclimatized for two to three weeks at 15-20°C under continuous LED light at 200 μmol m^-2^ s^-1^. During April, the plants were transferred to an unheated commercial greenhouse. Plants were maintained in the Jiffy 7 plugs until next spring (for one year) when all surviving plants were transplanted to peat pots (10 cm diameter). Plants were grown for two more seasons under natural light conditions. The temperature was kept above minus 5°C and below 25°C. During the growing season, potted plants were regularly watered and fertilized to keep soil humidity. Survival and height were recorded after the second and third growing seasons in the greenhouse.

### 2.4 Statistical analyses

Data were analyzed using the software package SAS^®^ 9.4 (SAS Institute Inc., 2013. SAS^®^ 9.4 Statements: Reference. Cary, NC: SAS Institute Inc.). A simple analysis of covariance using procedure GLM on measured data was applied using clone as fixed effect and temperature, initial embryo score and week of germination as covariates. Clone and temperature interaction was included for germination analyses. Other interactions were not significant. The germination analyses were based on mean of each Petri dish holding 20 embryos. For steps 1 to 3 single tree observations were used for data analyses. Again, clone was treated as fixed effect and different sets of covariates were included. A specific analysis of effect of box was carried out using procedure MIXED and box as random effect. No significant effect of box was seen for any of the traits, and box was therefore omitted from the model. Normality of error variance and homogeneity of error variance was checked by normality test and plotting residuals against predicted values using the SAS GLM option ‘plot=all’. No important deviations for assumptions of variance homogeneity and normal distributed errors were seen.

## 3 Results

### 3.1 Germination success

**Figure 3:**
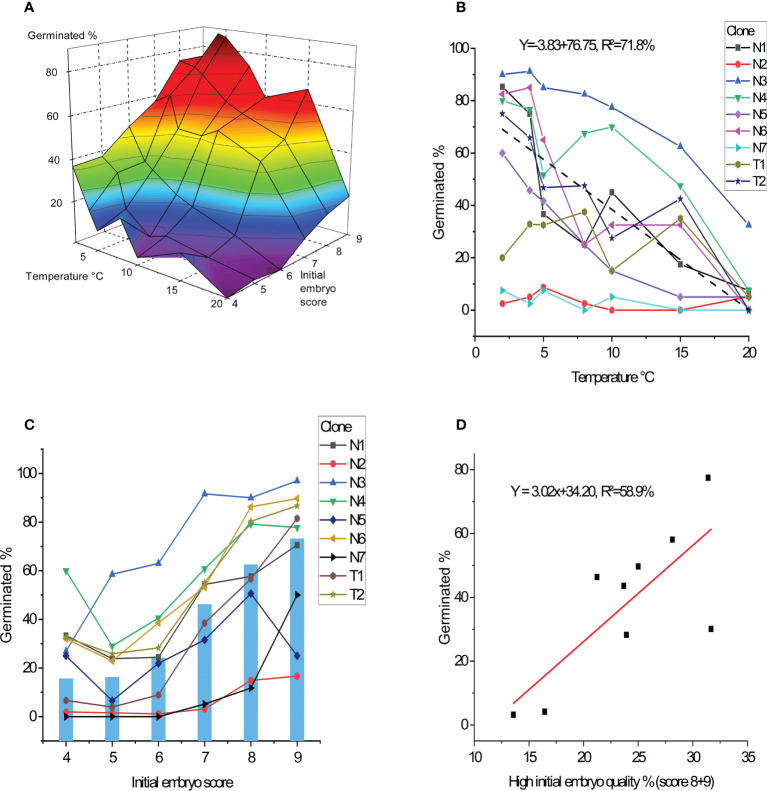
Percentage of germinated embryos after 11 weeks. **(A)** Average germination frequency in percentage across all nine clones in response to germination temperature and initial embryo quality score. **(B)** Average germination for each clone in percentage as response to temperature treatments (average performance of clones showed by dashed line). **(C)** Average germination for each clone in percentage as response to initial embryo quality score (average across clones showed in bars). **(D)** Average germination for each clone in percentage as function of frequency of percent high quality initial embryos (percent score 8 + 9).

After 11 weeks of germination, temperature, clone and initial embryo quality all had a strong impact on germination success rates (p<0.001; **Table 3**, **Figure 3A**). Average germination success rate across all clones and temperatures was 38%. Germination success rates decreased with increased temperature levels during germination (**Figure 3B**). The better the initial embryo quality, the higher the germination success rate (**Figure 3C**). An optimum of above 80% germination success rate was seen for embryos of high initial quality when germinated under lower temperatures at 2-5°C.

**Table 3:** Results from analyses of covariance for germination success, survival at the end of year 3 in the nursery, height after 2 and 3 years in the nursery and increments from previous year.

Trait		Temperature	Clone	Clone x temperature	Initial embryo score	Week of germination	LED light intensity
Germination*	DF	1	8	8	1		
	F-value	178.02	27.56	9.01	31.1		
	p-level	**<.001**	**<.001**	**<.001**	**<.001**		
Week of germination*	F-value	**36.35**	**4.04**	**1.60**	**1.32**		
	p-level	**<.001**	**<.001**	0.1324	0.2533		
ResuIts at end step 3 nursery							
	DF	1	6	**	1	1	1
Survival	F-value	16.67	14.09		7.19	5.74	1.98
	p-level	**<.001**	**<.001**		**0.007**	**0.017**	0.160
Height 2y	F-value	0.80	7.48		0.97	10.95	5.60
	p-level	0.372	**<.001**		0.326	0.001	0.019
Height 3y	F-value	0.15	14.03		0.25	19.38	4.59
	p-level	0.695	**<.001**		0.617	**<.001**	**0.034**
Increment height 2y-3y	F-value	1	14.52		1.27	19.69	2.89
	p-level	0.320	**<.001**		0.261	**<.00**1	0.091

**Not signifikant- removed from models.

* Based on mean of each petri dish. Effect of clone and the covariates temperature, interaction clone and temperature during germination, initial embryo score prior to germination, week of germination (week 8 to 11 from start of germination) and LED light intensity during the 8 weeks period of autotrophic growth on sugar free medium. Colors describes probability level (based on significance F-tests) for each factor or covariate. Bold values means significant <5% level.

No differences in the germination success rates could be detected between the two species *Abies nordmanniana* and *A. bornmuellerianna*, but strong differences between clones were seen and deviating clone patterns across temperatures (p<0.001; **Figure 3B**). The best clone (N3) had the highest germination success rate across all temperatures with a maximum at 92%, whereas the two poorest performing clones (N2, N7) had below 10% germination success rate despite temperature.

Evaluated across all clones, germination success rate related to initial embryo quality ranged from 16%, (score 4) to 73% (score 9) (**Figure 3C**). Rather large clonal differences in germination success rates were seen, although the clones showed a similar pattern in germination success rates across embryo scores (no significant interaction between clone and initial embryo score).

Clones included in this study showed different proportion of higher quality embryos (defined as scores 8-9), and this factor had a strong impact on embryo germination percentages accounting for 59% of the variation (**Figure 3D**). However, the two clones with above 30% of higher quality embryos had different germination percentages of 30% and nearly 80% respectively.

Number of germinated embryos was registered during week 8 to 11. Most embryos germinated in week 8 and thereafter in declining numbers (week 8: 724, week 9: 320, week 10: 109 and week 11: 21). No new germination was seen in week 12.

**Figure 4:**
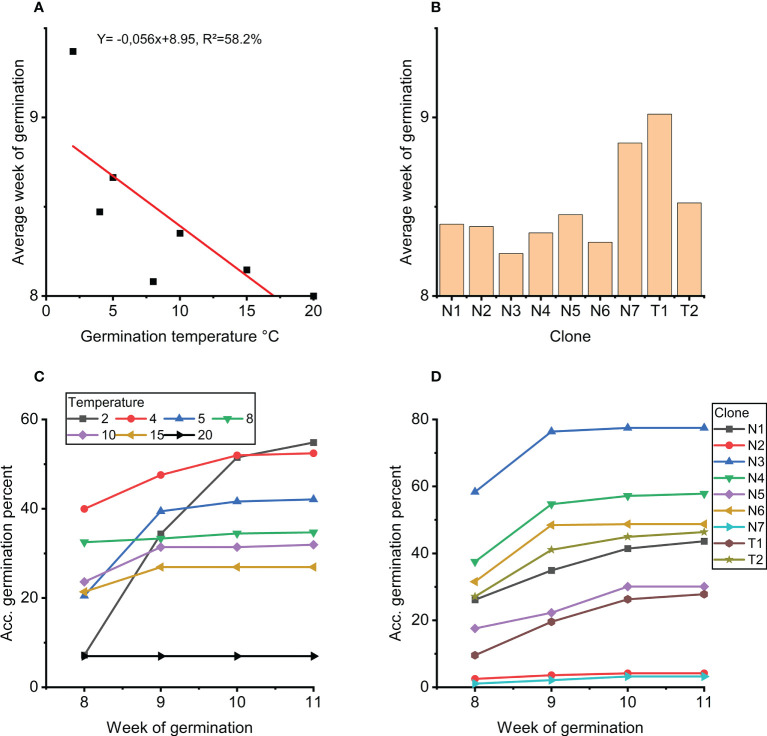
**(A)** Average week of germination as a function of germination temperature. **(B)** Average week of germination for each clone. **(C)** Accumulated germination in percentage for weeks 8 to 11 for each temperature treatment 2-20°C. **(D)** Accumulated germination in percentage for weeks 8 to 11 for each clone.

Analyses of covariance of week of germination showed that both germination temperature (**Figure 4A**) and clone (**Figure 4B**) strongly influence average week of germination (p<0.001; **Table 3**), and no clone and temperature interaction was found (p=0.123).

Further analyses of the strong effect of temperature on week of germination (week 8 to 11) show that germination at 20°C peaked already at 8 weeks with no further germination thereafter (**Figure 4C**). For germination at 2°C, germination accummulated over the weeks with some germination also in week 11. Germination at 4°C showed the highest level of germination after 8 weeks with continued germination in week 9 and 10. In week 11, limited germination was observed only at 2°C and 5°C.

Clones N3, N4 and N6 germinated fastest (**Figure 4B**). These clones also showed the overall highest accumulated germination, and no new germinants after week 9 (**Figure 4D**). In general, the order of the clones at week 8 and week 11, respectively, was the same from low to high percent accumulated germinated embryos, i.e. no rank changes (**Figure 4D**).

### 3.2 Survival of plants

Of the 3097 mature embryos started for germination, a total of 1174 mature embryos germinated after 12 weeks of culture. Genotypes N2 and N7 of *A. nordmanniana* were excluded from further analyses due to the low number of geminated embryos (9 and 15, respectively) resulting in a total of 1150 germinated embryos from seven clones for further analyses (**Tables S4, S5**).

All germinted embryos survived after the first 8 weeks of culture under different LED light treatments (step 1), but only 43.7% survived (499) after transplanting into plugs and an additional 8 weeks of growth under one intensity of LED light (step 2). Of the 499 plants successfully tranferred to the greenhouse, 159 suvived after 3 growing seasons corresponding to an overall greenhouse-mortality of 32%. Therefore, overall survival rate of plants from mature embryos was 13.8% (159/1150) across all clones and temperatures.

**Figure 5:**
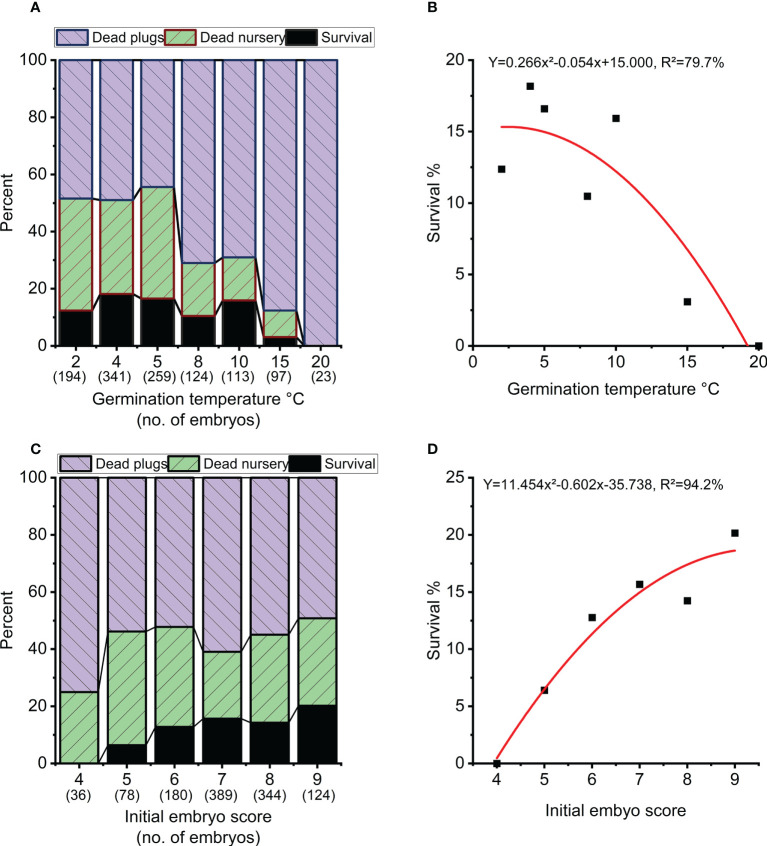
Survival in percentage of starting numbers of embryos at the end of nursery growth (black), mortality during the 8 weeks of plant growth in plugs (blue) and 3 years of plant growth in the nursery (green). **(A, B)** Overall survival as a function of temperature during germination. **(C, D)** Overall survival as a function of initial embryo score.

**Figure 6:**
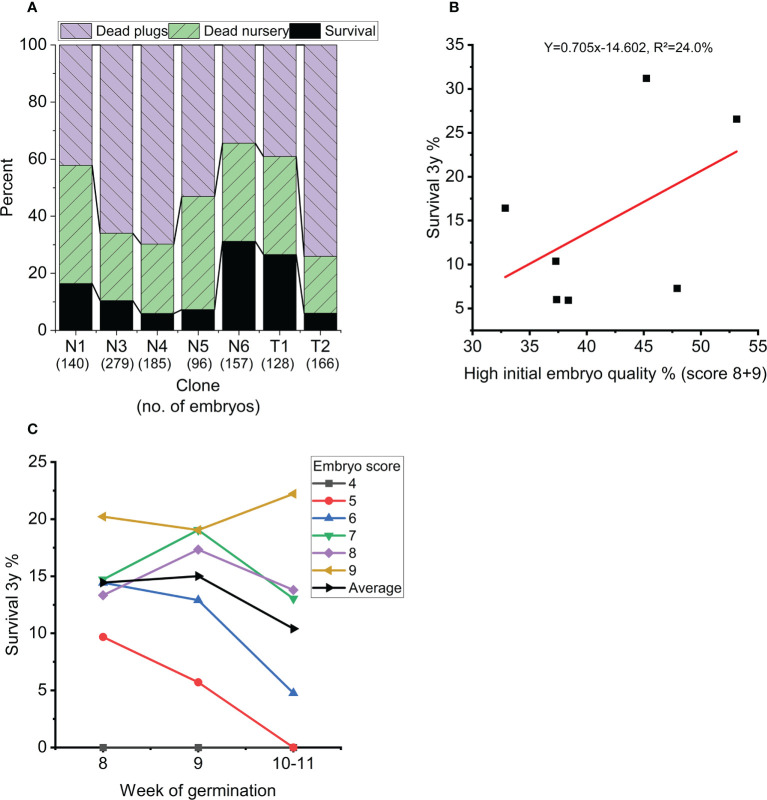
**(A)** Average survival for each clone in percentage at the end of nursery growth (black), mortality during 8 weeks of plant growth in plugs (blue) and 3 years of plant growth in the nursery (green). **(B)** Average survival of each clone (at the end of nursery growth) as a function of percentage high quality embryos (score 8-9) across all treatments. **(C)** Average survival across treatments at the end of nursery growth as a function of week of germination (dashed line) and for each initial embryo score (average across all clones and treatments). Weeks 10 and 11 are grouped together due to few observations.

Overall survival rate from mature embryo at end of the nursery stage was strongly impacted by germination temperature (p<0.001), initial embryo score (p=0.007), clone (p<0.001) and to a lesser extend week of germination (p=0.017) (**Table 3**, **Figure 5**). The optimal germination temperature for survival was 4°C (**Figures 5A, B**). Survival rates inreased with embryo quality varying between 0% for embryo score 4 to 20% for embryo score 9 (**Figures 5C, D**). Clones ranged in survial rates from 6% (N4) to 31% (N6) (**Figure 6A**). This difference can partially (24%) be explained by differences in proportion of high quality embryos observed among clones (**Figure 6B**). For week of germination, survival was lower for the last germinating embryos from weeks 10 and 11 (**Figure 6C**). There seems to be a tendency (**Figure 6C**) for higher initial quality embryos (scores 7-9) to be relatively better performing across harvest weeks compared to poor quality embryos (scores 4-5), although this interaction between initial embryo score and week of germination is not significant (p=0.120).

### 3.3 Growth in the nursery

**Figure 7:**
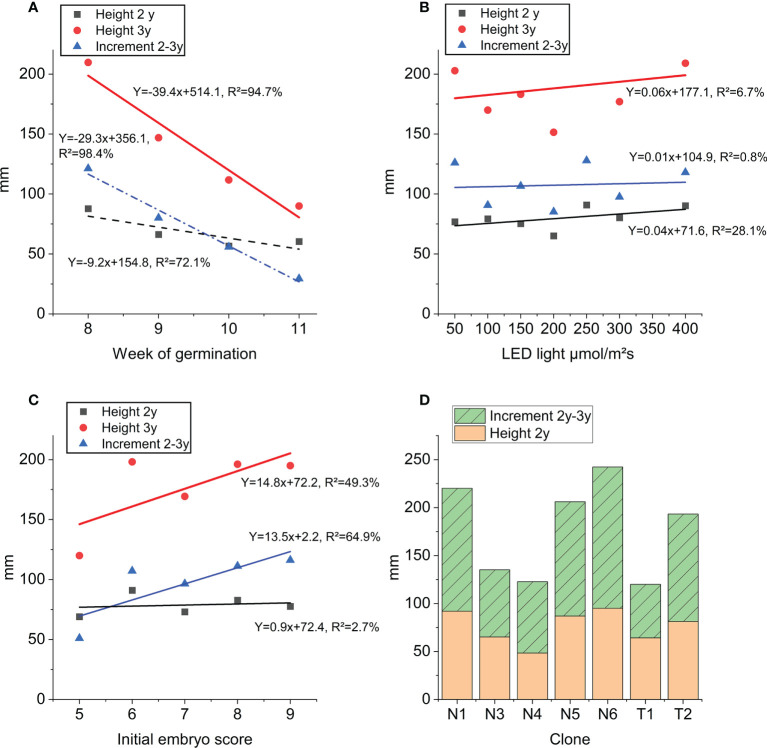
Height growth at end of step 3 nursery plant growth: 3-year height (red), 2-year height (black) and height increment (blue): **(A)** as function of week of germination, **(B)** as function of LED light intensity (applied in step 1), **(C)** as function of initial embryo score prior to germination (averages across all clones). Trendline equations and explanation rate (R²) for each curve and trait. **(D)** Average height of clones (mm) across all treatments: orange 2-year height and green increment 2-year to 3-year and stacked bars 3-year height.

The average height of plants after 3 years in the nursery was 17.8 cm. Week of germination registered three years earlier had a strong impact on 2- and 3-year heights as well as height increment during last year in the nursery (**Figure 7A**). The linear relationship explains 72-98% of the variation in height across germination weeks. Although significant, LED light intensity (step 1) only had very little impact on 2- and 3-year heights measured in the nursery (**Figure 7B**). There is a strong linear relationship between nursery 3-year height growth and initial embryo quality score describing 49% of the variation in height (**Figure 7C**, **Table S5**). Similarly, initial embryo quality could describe 65% of the variation in 2-3 year height increment. Mean values across clones for 3-year height growth ranged from 12.3 cm (T1) to 24.3 cm (N6) (**Figure 7D**).

### 3.4 Cumulative effects across steps

**Figure 8:**
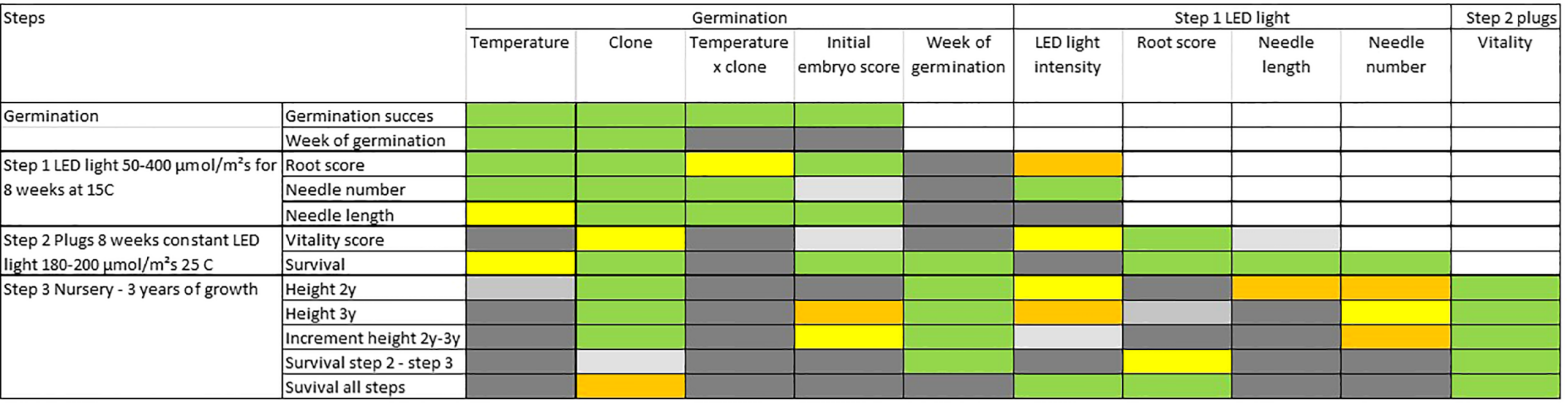
Results from statistical analyses of covariance (ANCOVA) describing how important each effect is for the trait of interest during steps 1 to 3. Colors describes probability levels (significance) of F-tests for each factor or covariate: Green <0.1%, orange 0.1-0.99, yellow 1-4.99%, light grey 5-10%, grey >10%.

An overall analysis of covariance, at each step adding the previous results as covariates, reveals a strong interrelation between consecutive steps and treatments, and for measured (or scored) characteristics (**Figure 8** and **Table S5** for detailed statistical results). Across most developmental steps and measured traits, clones as well as initial embryo score have a significant influence on the results (**Figure 8**). Week of germination has a relatively strong impact on survival in plugs (step 2) as well as on survival and height-growth in the nursery (step 3), but no effect during the earlier growth in aerated boxes (step 1).

The intensity-level of LED light (during step 1) have a minor although significant impact on several traits (**Table S5**). Most significant is the positive effect on root score (step 1) with an optimum at about 100 µmol m^-2^ s^-1^ and decreasing effects with increasing light intensities (**Figure S5**). For the number of needles, a light intensity above 50 µmol m^-2^ s^-1^ at 100-200 µmol m^-2^ s^-1^ seemed optimal. Only minor differences in the impact on traits were seen for higher light intensities (**Figures S3, S4**).

**Figure 9:**
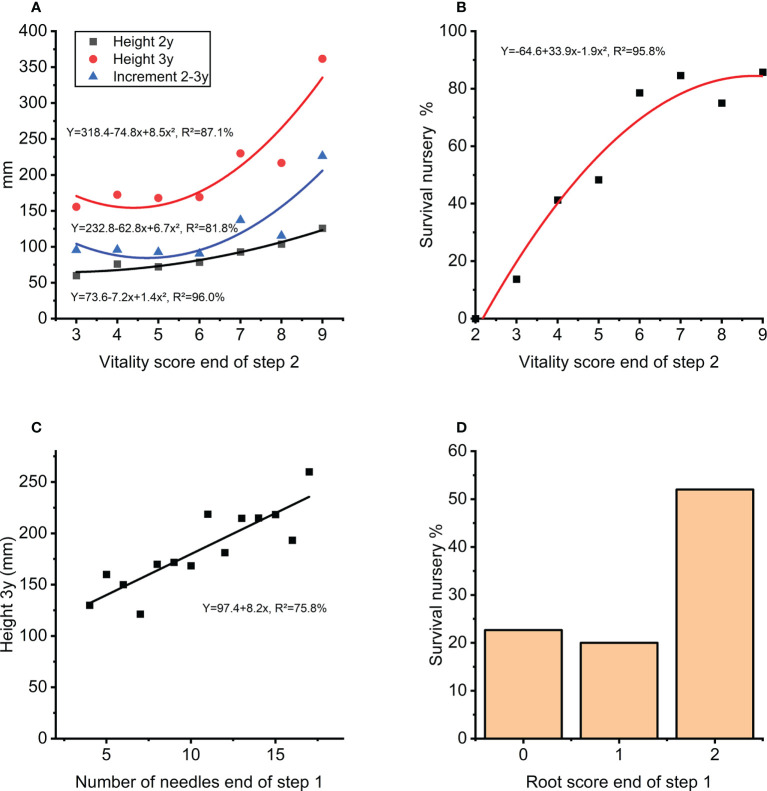
**(A)** Height growth in nursery (step 3) as function of vitality score (step 2). **(B)** Survival in percentage in the nursery (during step 3) as a function of vitality score (step 2). **(C)** Height after 3-years in the nursery (step 3) as a function of number of needles at end of first 8 weeks of autotrophic growth (step 1). **(D)** Survival in the nursery in percentage (during step 3) for score classes of root development at the end of first 8 weeks of autotrophic growth (step 1).

The traits measured at the end of step 1 (rooting, needle-length and needle number) had a positive correlation to survival in step 2. The score for plant vitality at the end of step 2 arguably depends on the effect from rooting and needle-numbers in the previous step and was also a good indicator for height growth and survival in the next nursery step three years later (**Figure 9A**) describing 87% and 96% respectively of the average height growth and survival at the nursery stage during step 3 (**Figure 9B**). Interestingly, the number of needles at end of step 1 can explain 76% of the variation in nursery height growth at step 3 (**Figure 9C**). A positive effect of high root score can also be seen in survival during this stage (**Figure 9D**).

## 4 Discussion

### 4.1 Opportunities for SE as a tool for conifer plant production

The SE process for plant production in conifers is a row of consecutive and interrelated steps starting under sterile conditions in the laboratory continuing in a growth chamber or greenhouse. Losses of plant materials are expected in each step but to different degrees largely depending on genus. SE in *Picea* and *Larix* are overall successful for most species, whereas for *Pinus* the SE process is less successful and often supported by cutting technology applied to the SE plants as a second step to boost plant numbers (Klimaszewska et al., 2016). Less studies have focused on *Abies* species however Find (2016) reported on the growth of nine *Abies nordmanniana* somatic clones under field conditions after 8 growing seasons in the field. In Germany, two SE-clones have been grown to final size for Christmas trees (Blödtner-Piske, 2017). For the approximately 40 other *Abies*-species (Liu, 1971; Farjon and Rushfort, 1989) successful multiplication and plant generation by SE has only been shown for *A. lasiocarpa* (Kvaalen et al., 2005), *A. cephalonica* (Krajňáková et al., 2009), *A. alba* (Hristoforoglu et al., 1995; Salaj et al., 2020), *A. fraseri* (Pullman et al., 2016), and several hybrids (Salaj et al., 2019). Plants growing ex vitro have typically only been obtained in small numbers and due to the low number of propagules for the later stages of development, few studies have focused on germination and plant growth in any *Abies*-species.

In the present study, factors effecting growth of SE-derived plants of *A. nordmanniana* and *A. bornmuelleriana* were studied starting from the mature embryo-stage through the stages of germination, acclimatization, and growth during three seasons ex vitro.

### 4.2 Effect from cryostorage on embryogenic culture performance

The embryogenic cultures used in the study were either thawed from cryogenic storage (*A. nordmanniana*) or used for the experiments directly after initiation from fresh, immature seeds (*A. bornmuelleriana*). There are to date no reports showing that cryogenic storage affects plant production in a significant way and the successful protocol for cryopreservation of *A. nordmanniana* SE cultures reported early on (Nørgaard et al., 1993) is still in use (Nielsen, pers. comm.). The effect from cryo-storage on proliferation of embryogenic cultures has been specifically studied in embryogenic cultures of hybrid firs (*Abies alba × A. cephalonica, Abies alba × A. numidica*) but no negative effect from cryopreservation was found (Salaj et al., 2016). In the present study, the pre-treatments of the embryogenic cultures were specific to each species, and there is therefore a strong confounding effect on the potential effect from cryo-storage. We were however not able to detect any differences that could be related to species and therefore cryostorage (data not shown).

### 4.3 The key developmental step of embryo maturation

Obtaining a high yield of mature embryos that can germinate and form viable plants is still a major bottleneck for most conifers and an obstacle for cost-effective up-scaling of SE plant production. The morphologies of the somatic embryos at the end of the maturation treatment typically vary widely within the same culture batch due to the lack of synchronization during development. Such morphological differences are critically related to the yield of mature embryos with germination capability, as only embryos that have reached the responsive developmental stage of maturation can continue to germinate. The lack of synchronization during maturation is believed to be an important factor for low maturation yields and has prompted efforts to optimize methods for synchronization (Jamruszka and Jamruszka-Lewis, 2009; Gupta et al., 2011; Mamun et al., 2018). Both physiological and cultural factors that have been associated to mature embryo quality and yield could at least partially explain the lack of synchronization. During the conifer SE developmental process, arguably the developmental steps are different and inferior to the corresponding processes in the zygotic embryo simply based on the fact that the somatic embryo obtains nutrient supply from the culture media versus the zygotic embryos feeding from the endosperm and it is challenging to create an exact match of the natural seed environment. In support of this view, based on biochemical analyses it appears that mature embryos of *Pinus pinaster* get arrested at an early developmental stage of maturation and never reach the same developmental stage as their fully mature zygotic counterparts (Morel et al., 2014) implying that germination may overall not be as successful in a somatic embryo as in a zygotic embryo. Furthermore, the lack of synchronized maturation can be traced back to the earliest stages of somatic embryos in the PEM culture that does not multiply in a synchronized way resulting in cultures composed of PEMs with different capabilities to respond to the maturation treatment (Filonova et al., 2000). The relative presence of the most developed, maturation-responsive PEMs at the transition to maturation will thereby determine the yield of mature embryos. Dispersion of PEM cultures before maturation increases medium access and allowed for a larger number of PEMs to respond to maturation treatment, resulting in an overall improved synchronization of maturation response in *Picea abies* (Mamun et al., 2018). The positive effect on maturation yields from polyethylene glycol (PEG) added to the culture medium is well established (Stasolla al. 2002). However, subsequent germination frequencies have also been noted to be significantly lower after maturation on a medium containing PEG (Stasolla et al., 2002; Businge et al., 2013 and others). In *Abies* species, inclusion of PEG in the culture medium resulted in successful maturation and formation of higher yields of mature embryos in hybrid *Abies* species (Salajová and Salaj, 2001; Salaj and Salaj, 2003; Salaj et al., 2004; Salaj et al., 2005), *A. numidica* (Vooková and Kormuták, 2002), *A. cephalonica* (Krajňáková et al., 2009), *A. alba* (Salaj et al., 2020) and *A. nordmanniana* (Nørgaard, 1997). PEG was also included in the maturation medium in the present study and supported the formation of mature embryos with germination potential. The proportion of mature embryos displaying more advanced morphologies that can continue to develop to germination and plant formation in effect corresponds to the degree of synchronization of a culture and is closely related to clonal identity as was also noted in the present study (**Figures 3B, C**). Here the effect of embryo morphology (initial embryo score) could be traced throughout development to the third year of growth (**Figures 7C**, **8**). Furthermore, week of embryo germination had a strong effect on three-year height growth (**Figure 7A**). Results from germination of zygotic seeds of *A. nordmanniana* also indicate a negative effect from late germination on seedling growth even after two years (unpublished data). This could be an effect of the shortened first growing season but also just reflect less viable seeds causing the delay in germination. Fast germination could therefore be an additional quality trait to consider when scoring for SE capabilities of clones.

### 4.4 Temperature effects on germination

Chilling influences both the depth of the mature seed’s primary dormancy and later stimulates dormancy breaking (Batlla and Benech-Arnold, 2010). The importance of temperature during seed development for the control of timing and success of the breaking of dormancy and in effect start of germination was also demonstrated in *Pinus contorta* var. latifolia and *Picea glauca* (Moench) Voss x *Picea engelmannii* Parry ex. Engelm. and *Tsuga heterophylla* (Liu and El-Kassaby, 2015). Further studies showed that seed dormancy and germination was correlated with changing levels of endogenous ABA, GA, and auxin and specifically a modulation of interactions between central auxin-signaling pathway components (TIR1/AFB, Aux/IAA and ARF4; Liu and El-Kassaby, 2015). It was previously demonstrated that most if not all *Abies* species go through seed dormancy (Baskin and Baskin, 2014) and respond to stratification with increased and elevated germination rates (Edwards, 2003). The stratification temperature was shown to affect germination percentage and mean germination time in *Abies marocana* (Hatzilazarou et al., 2021). The result from the present study shows that temperature during germination is determining the success rate (**Figures 3A, B**) and time of germination (**Figure 4A**) also for somatic embryos of *A. nordmanniana* and *A. bornmuelleriana* with an optimal temperature for germination at 4°C (**Figure 3A**). Similarly, somatic embryos of *A. cephalonica* were desiccated at high relative humidity at 4°C for three weeks before being transferred to germination (Krajňáková and Häggman, 2016). Cold storage at 4°C before germination was also applied to *P. abies* somatic embryos (Tikkinen et al., 2018b), and *A. nordmanniana* somatic embryos have been successfully vernalized at 7°C (Nawrot-Chorabik, 2016).

### 4.5 The effect of LED light intensity during germination

Light sources used in greenhouses and for indoor production of plants in general can be described by a number of characteristics, e.g. intensity (µmol m^-2^ s^-1^), quality or color (wavelength in nm) and direction and duration, which in numerous ways impact plant physiology and secondary metabolism (Ouzounis et al., 2015). Zygotic tree seedling growth can be manipulated by changing wavelength and intensity, but responses varies between species, root versus top, and developmental stage of the seedling (e.g. Montagnoli et al., 2018; Navidad et al., 2020). The light-quality has been shown to have a strong influence on the success of germination of somatic embryos in *Picea abies* such that blue light seemed to broadly inhibit germination processes whereas red light acted stimulatory for the germination process (Kvaalen and Appelgren, 1999). Germination from seeds were however not notably affected by the different light qualities in these experiments.

LED lights of different wavelength have been tested showing that white light (400-700 nm) was the best compared to blue, red and far-red light for germination of somatic embryos in *A. nordmanniana* (Nawrot-Chorabik, 2016). In another study on *Picea abies* germination, LED lights were tested at 2500K (Valoya L14 spectrum AP67 Milky LED, Valoya Oy, Helsinki, Finland) from 5 to 150 µmol m^-2^ s^-1^ together with other factors during germination, however a specific effect from the light intensities could not be identified (Tikkinen et al., 2018b). For optimal height growth in the greenhouse, it was concluded that 150 µmol m^-2^ s^-1^ was less effective than the higher rates (Tikkinen et al., 2018a) suggested from previous studies (Landis et al., 2010; Rikala, 2012). Varis et al., 2021 found little effect of low-intensity LED lights (Valoya L14 spectrum AP67) during proliferation, and at the end of maturation. Germination of *P. abies* was later shown to be successful under an intensity of 190–210 µmol m^-2^ s^-1^ (Valoya L14 spectrum AP67 Milky LED, Valoya Oy, Helsinki, Finland) with an 18 h/6 h day/night photoperiod (Välimäki et al., 2021). These studies agree with our study using white LED light (4000K) after germination for photoautotrophic growth where low intensities seemed to have little effect and best results were seen for 100-200 µmol m^-2^ s^-1^. This was also found to be optimum for growth of zygotic *Picea abies* seedlings after testing light intensities from 50 to 400 µmol m^-2^ s^-1^ (Velasco and Mattsson, 2019). A light intensity level of 100-200 µmol m^-2^ s^-1^ is also used for numerous species in greenhouse production (Ouzounis et al., 2015). In our study growth under continuous light (24h) was applied for the first 8 weeks of photoautotrophic growth to stimulate increased growth as was previously demonstrated for *Picea pungens* seedlings grown under continous light (Young and Hanover, 1978). However, a long-day treatment could affect growth cessation and annual growth rhythm (Ekberg et al., 1979) causing problems for acclimatization when moved to the greehouse for final plant development.

### 4.6 Practical implications from cumulative effects on SE plant growth

The cumulative effect of consecutive steps of SE plant production has been described for *P. abies* (Högberg et al., 2003). It was found that epicotyl length and presence of lateral roots at time of *ex vitro* transfer were important selectable traits for obtaining an overall more homogenous plant material. Also, cold storage of embryos, a low nitrogen content in the germination medium and limited time for *in vitro* germination (one week) were factors shown to have a strong positive effect on plant growth and survival in *P. abies* (Tikkinen et al., 2018b).

The concept of selection at different production steps to optimize growth and quality is well known from commercial bare-root nursery practices for zygotic plants. Selection procedures include sorting of seeds to discard empty and small seeds prior to sowing, sorting of two-year old seedlings before transplanting, and finally, sorting of 3-year or 4-year plants by a minimum height or collar diameter depending on buyer demands.

By applying the same principle to SE plant production, initial embryo quality would resemble seed quality. Although initial embryo quality was evaluated in the same way for all clones and there was a strong general trend of improved germination with higher initial embryo quality scores, clones showed large differences in successful germination even from similar initial embryo scores (**Figure 3C**) and for similar proportions of high-quality embryos (**Figure 3D**). This implies that the visual appearance of the embryo prior to germination can be used for selection for successful germination, however the specific clone performance also has to be considered. The best embryo is the best only within the clone since the overall performance will largely depend on the specific clone. Additionally, the early-germinated embryos seem to have the best subsequent development irrespective of embryo score and clone (**Figure 6C**).

A central step is moving plants in plugs to the commercial nursery (*in vitro* step 2 to ex vitro step 3; **Figures 2** and **8**). Only transferring fully green plants having at least five needles or above (vitality score 6+; **Figure 9B**) will result in a survival rate of close to 80% after the nursery step. Similarly, number of needles at end of the first part of *in vitro* autotrophic growth (step1) was strongly related to height-growth (step 3; **Figure 9C**). Both observations indicate that the number of needles is a strong trait to use for selection for further successful cultivation. A stepwise selection procedure potentially helps to lower production costs and to overcome excess variation within clones, and also limits the final need for sorting/selection of the commercial plants as was suggested previously for *P. abies* (Högberg et al., 2003).

In the present study, across all steps and traits measured, clones have a strong influence on the general performance of SE-based plant propagation. In *P. abies*, both family and genotype within family strongly affect the propagation success and determine how many genotypes that can be successfully initiated and grown into plants (Högberg and Varis, 2016). Differences in growth and survival between *P. abies* SE clones have also been described (Högberg et al., 2003; Tikkinen et al., 2018a; Tikkinen et al., 2018b). In our study, clones showed differences in nearly all steps starting from the initial embryo quality, proportion of high-quality embryos, germination, and finally survival and height after three years of nursery growth. Our data is however too limited to document family and species differences but clearly demonstrate the clonal differences. Optimizing protocols for single clones might be possible, however the strong clonal variation in the potential to multiply and mature PEMs, germinate mature embryos and establish and grow plant arguably reflects many multi-gene effects within the clones and therefore optimization for single clones could be difficult. A pragmatic approach is simply to work with the ‘easy’ clones for SE plant propagation (Högberg and Varis, 2016). However, for long-rotation forest tree species, planting of selected clonal families pose a problematic risk of losing genetic diversity if successful numbers within families are too skewed (Rosvall et al., 1998) whereas for short rotation species like Christmas trees when clone testing is applied, the risk is less. In Denmark, for *A. nordmanniana* and partly *A. bornmuelleriana*, the application of SE for commercial use is already underway with clonal field trials established in 2014-2015 and 2019-2021 holding a total of approximately 625 clones (Find, 2016; Nielsen, 2022, unpublished data). A ‘superior’ Christmas tree combines numerous characteristics (Nielsen et al., 2020) e.g., crown symmetry, needle orientation, steady growth, numerous branches, postharvest needle retention, fungus, and insect resistance that are all measured during scheduled evaluations.

## 5 Conclusion

There is strong clone effect present in most of the traits measured as important for the SE propagation process. Furthermore, most traits evaluated during germination and growth not only influence the nearest consecutive step, but some also impact growth and survival three years later. This highlights the importance of viewing the production of SE plants as a consecutive row of strongly interrelated steps. Selection based on key-traits during the developmental process can be an important tool to improve quality as well as economy by saving time needed for handling and demand for space. A pragmatic approach for improving *A. nordmanniana* SE production could be to first select clones with known, good SE performance, only use high quality embryos (score >7), germinate at 4°C, discard embryos germinated after 10 weeks and further, only transplant plantlets having at least a 2 mm white root and five needles, and at time of transfer to greenhouse discard plants of poorer vitality (<score 6). Optimizing embryo quality by improved methods for cultivation and better understanding of selection criteria are attractive targets for further investigation.

## Data availability statement

The raw data supporting the conclusions of this article will be made available by the authors, without undue reservation.

## Author contributions

UN: participated in organizing, planning and design of the experiment, developing the initial embryo score, statistical analyses and writing of manuscript. CH: participated in developing the initial embryo score, maintaining the cultures and maturating the embryos, setup of cold treatments and did all scorings of initial embryo quality, and evaluation of embryos during week 8-12, and commented the manuscript. UH: conducted all the culturing during the nursery stage and commented the manuscript. VJ: participated in organizing the project, planning and commented the manuscript. UE: participated in interpreting analyses and writing of the manuscript. All authors contributed to the article and approved the submitted version.

## Funding

The project was partly financed by the Green Development and Demonstration Program, grant 340009_16_1081, Danish Agricultural Agency – years 2016-2021.

## Acknowledgments

The substrates and media used in this study was based on most recent procedures for somatic embryogenesis for *Abies nordmanniana* developed by late Jens I. Find and colleague’s gardener El Birhmann and lab technician Lisbeth Hansen. Thanks to El Birhmann for describing procedures of transplanting to plugs. Special thanks to Lisbeth Hansen for preparing substrates and media, initiating cell lines, maturing the embryos and follow the experiments with such a precision, care and knowledge. Furthermore, for providing initial information on presumed embryo quality when developing the initial embryo score. Thanks to Jesper Riis Christiansen for introducing the idea of using simple commercial refrigerators in combination with an external temperature control, and to Mads Madsen Krag for introducing and installing fans to homogenize air temperature within the refrigerators.

## Conflict of interest

The authors declare that the research was conducted in the absence of any commercial or financial relationships that could be construed as a potential conflict of interest.

## Publisher’s note

All claims expressed in this article are solely those of the authors and do not necessarily represent those of their affiliated organizations, or those of the publisher, the editors and the reviewers. Any product that may be evaluated in this article, or claim that may be made by its manufacturer, is not guaranteed or endorsed by the publisher.
