# Supplementary material for: Accumulated effects of factors determining plant development from somatic embryos of Abies nordmanniana and Abies bornmuelleriana
Source: Front Plant Sci. 2022 Oct 13;13:989484. doi: 10.3389/fpls.2022.989484 (PMC9608518; doi:10.3389/fpls.2022.989484)
Supplement: Supplementary file 1 [file DataSheet_1.pdf]

Supplementary material – tables and figures:

Table S1. Vitality score at end of step 2 after eight weeks of growth under continuous LED light.

Category	Vitality score
**Good and vigorous plants**	9 = green needles – often more than 10 needles, shoot extended or long needles
	8 = green needles - often more than 10 needles, maybe new needles
	7 = green needles – often 6-9 needles, maybe new needles emerging
**Acceptable plants**	6 = green needles, mostly 5 needles, maybe new small emerging needles
	5 = green needles, often shorter 3-5, maybe new small emerging needles or a good bud
	4 = green or yellowish needles, often 3 needles
**Poor plants**	3 = green parts on needles (max. 50%) – rest yellowish or brown
	2 = brown needles only bits of yellow or green

Table S2. Number of germinated embryos for each clone and germination temperature. Germinated embryos were transferred to step 1 and included in further studies.

**Clone**	**Temperature °C**							
	**2**	**4**	**5**	**8**	**10**	**15**	**20**	**Total**
**N1**	35	45	22	10	18	7	3	140
**N3**	36	73	68	33	31	25	13	279
**N4**	32	46	30	27	28	19	3	185
**N5**	24	27	25	10	6	2	2	96
**N6**	30	51	39	10	13	13	0	156
**T1**	7	46	38	15	6	14	2	128
**T2**	30	52	37	19	11	17	0	166
**Total**	194	340	259	124	113	97	23	1150

Table S3. Number of germinated embryos for each embryo score and germination temperature. All embryos were transferred to step 1 and included in further studies.

Initial embryo score	Temperature °C							
	2	4	5	8	10	15	20	Total
4	15	8	5	4	1	3	0	36
5	22	28	16	3	5	3	1	78
6	39	52	45	20	16	8	0	180
7	55	114	81	42	46	41	10	389
8	48	103	81	44	32	28	8	344
9	15	35	31	11	13	14	4	123
**Total**	194	340	259	124	113	97	23	1150

Table S4. Clone mean values for no. of plants germinated: Average initial embryo score of those germinated, higher quality embryos %, root score, white roots % (score 2), number of needles at end of step 1, length longest needle end of step 1, and dead plugs after end of step 2.

Clone	No. Plants	Initial embryo scoe	Higher quality embryos	Root score	White roots	Number of needles	Longest needle	Dead plugs after 8weeks
		mean score	score 8+9 %	mean score	score 2 %		mm	%
**N1**	140	7,1	33%	0,63	18%	5,3	8,6	42%
**N3**	279	7,1	37%	0,73	25%	3,9	6,9	66%
**N6**	157	7,1	45%	0,92	25%	6,3	9,8	34%
**N4**	185	7,2	38%	0,33	6%	5,1	9,0	70%
**N5**	96	7,3	48%	0,69	21%	4,3	7,4	53%
**T1**	128	7,5	53%	0,92	38%	5,8	8,2	39%
**T2**	166	6,9	37%	0,92	25%	4,0	7,9	74%

Table S5.

SUPPLEMENTARY material for Figure 8. Specific results from statistical analyses.

Results from statistical analyses of covariance (ANCOVA). Germination in percent.

Step 1: growth under different LED light treatments from 50-400 µmol/m^2 during eight weeks of autotrophic growth, traits measured at end of treatment: root score, needle number and length of longest needle.

Step 2: Transplanting into peat plugs and eight weeks of growth at 25 C under constant LED light – traits measured at end of period: survival and vitality score.

Step 3: growth in nursery during three growing seasons. Traits measured at end of period 2-year and 3-year height, increment and survival.

Effect of clone and covariates temperature, interaction clone and temperature during germination, initial embryo score prior to germination, week of germination (week 8 to 11 from start of germination) and LED light intensity during the 8 weeks period of autotrophic growth on sugar free medium, root score, needle length and length of longest needle and vitality score. Colors describe probability level (significance) of tests for each factor or covariate. Columns to the right DF: degrees of freedom, MSE: mean square error and Mean: overall mean value.

Steps			Germination					Step 1 LED light				Step 2 plugs			
			Temperature	Clone	Temperature x clone	Initial embryo score	Week of germination	LED light intensity	Root score	Needle length	Needle number	Vitality	DF	MSE	Mean
Germination*	Germinated (0/1)	DF	1	8	8	1							154*	0.0168	0.380
		F-value	178.02	27.56	9.01	31.1									
		p-level	<.001	<.001	<.001	<.001									
	Week of germination*	F-value	**36.35**	**4.04**	**1.60**	**1.32**							131	0.2305	8.51
		p-level	<.001	<.001	**0.1324**	**0.2533**									
* based on petri dish means values															
Step 1 LED light 50-400 for 8 weeks at 15C	Root score	DF	1	6	6	1	1	1					1144	0.5483	0.72
		F-value	60.93	6.59	3.02	10.58	0	5.84							
		p-level	<.001	<.001	0.006	0.001	0.949	0.016							
	Needle number	F-value	49.16	13.4	4.34	3.65	0.07	20.28					1150	6.86	4.9
		p-level	<.001	<.001	<.001	0.056	0.793	<.001							
	Needle length	F-value	8.22	6.08	8.07	12.75	1.21	0.91					1150	8.61	8.1
		p-level	0.004	<.001	<.001	<.001	0.271	0.342							
Step 2 Plugs 8 weeks contant LED light 25 C	Vitality score	DF	1	6	6	1	1	1	1	1	1		499	1.9051	3.86
		F-value	0.3	3.6	0.68	2.96	0.14	7.58	19.41	2.87					
		p-level	0.586	0.002	0.664	0.086	0.709	0.006	<.001	0.091					
	Survival	F-value	9.73	4.39	0.74	19.55	21.25	1.04	28.06	211.7	29.11		1144	0.1251	0.437
		p-level	0.002	<.001	0.617	<.001	<.001	0.309	<.001	<.001	<.001				
Step 3 Nursery - 3 years of growth	Height y2	DF	1	6		1	1	1	1	1	1	1	168	514.32	79
		F-value	3.52	6.41		0.12	15.57	6.92	2.34	5.06	6.49	36.61			
		p-level	0.063	<.001		0.730	<.001	0.009	0.128	0.026	0.012	<.001			
	Height 3y	DF	1	6		1	1	1	1	1	1	1	159	2480.98	184
		F-value	1.10	14.32		4.18	23.78	6.78	3.07	0.38	8.21	50.75			
		p-level	0.296	<.001		0.043	<.001	0.010	0.082	0.540	0.005	<.001			
	Increment height 2y-3y	DF	1	6		1	1	1	1	1	1	1	159	1267.90	104
		F-value	0.15	14.18		7.20	20.99	3.29	1.61	0.22	6.61	37.69			
		p-level	0.704	<.001		0.008	<.001	0.072	0.207	0.643	0.011	<.001			
	Survival step 2 - step 3	DF	1	6		1	1	1	1	1	1	1	499	0.1527	0.320
		F-value	0.10	1.89		0.35	11.05	0.00	8.28	0.53	0.98	114.68			
		p-value	0.750	0.081		0.554	0.001	0.997	0.004	0.467	0.322	<.001			
	Suvival all steps	F-value	1.04	2.74		0.00	0.67	22.07	13.64	1.45	1.92	357.12	1144	0.075	0.142
		p-value	0.307	0.012		0.984	0.413	<.001	<.001	0.228	0.166	<.001			

Supplementary material additional figures step 1.

Step 1 – photoautotrophic growth under continuous LED light (24) at different light levels. After 8 weeks of photoautotrophic growth each plant was measured. During transplanting into plugs – each batch of plantlets were shortly displayed on a table aside a ruler and a photo taken. Based on photo needles were measured and counted.

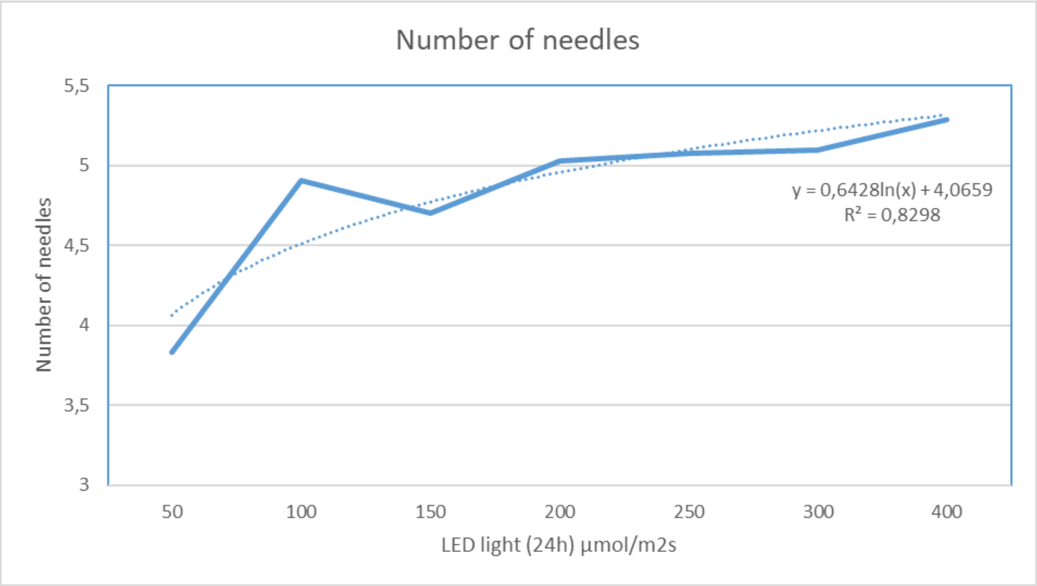

Figure S1. *Number of young needles after 8 week at 15 degree C and light intensities from 50 to 400 µmol/m²*.

Comment: As long light is kept above 100 µmol/m²s the differences in number of cotyledons are rather small.

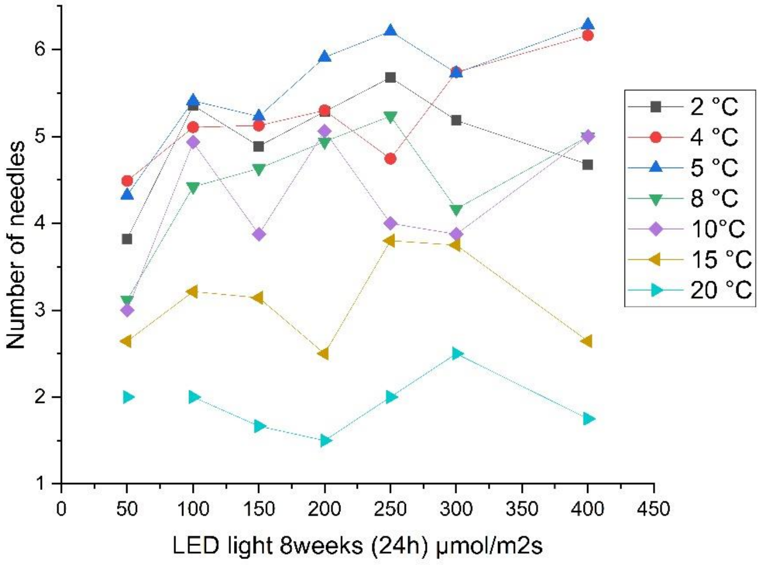

Figure S2. *Number of young needles after 8 week at 15 degree C and light intensities from 50 to 400 µmol/m². Lines describing performance of germinated embryos from temperatures 2-20°C.*

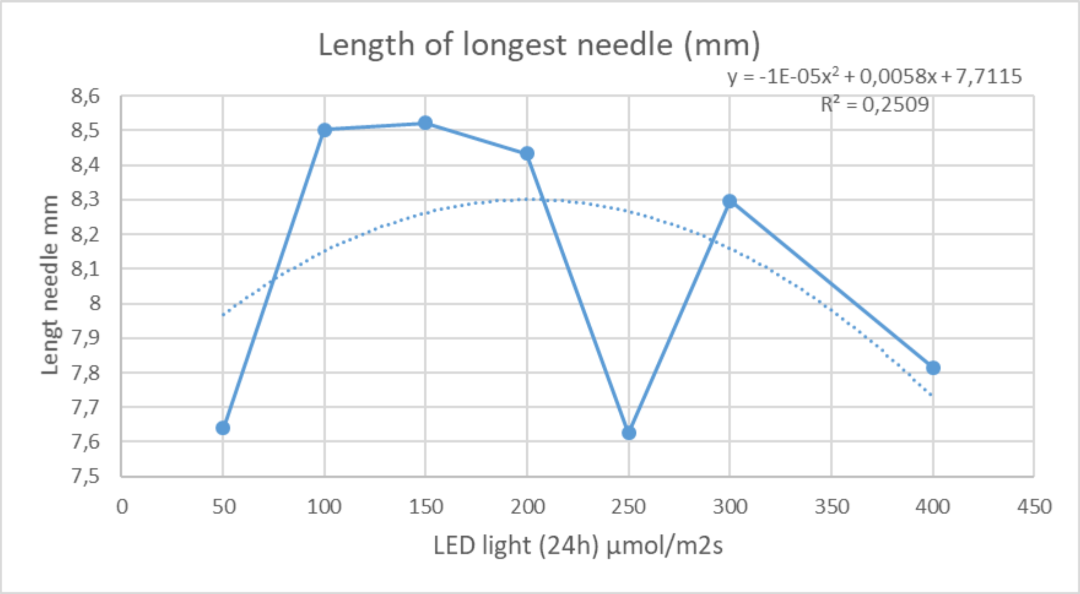

Figure S3. *Length of longest young needles (mm) after 8 week at 15 degree C and light intensities from 50 to 400 µmol/m^2.*

Comment: The length of the needles seems to have a maximum around a light intensity of 100 to 200 µmol/m^2.

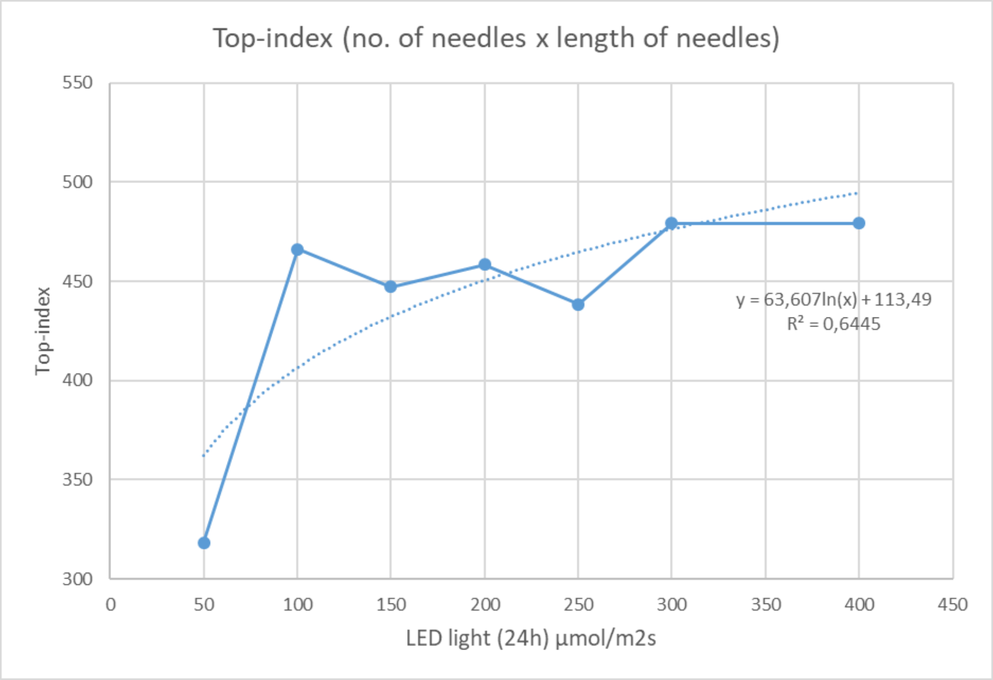

Figure S4. *Top-index (length of longest young needles, mm, multiplied numbers of needles) after 8 week at 15 degree C and light intensities from 50 to 400 µmol/m.^{2}*

Comment: As long light is kept above 100 µmol/m^{2}s the differences in topindex is rather small.

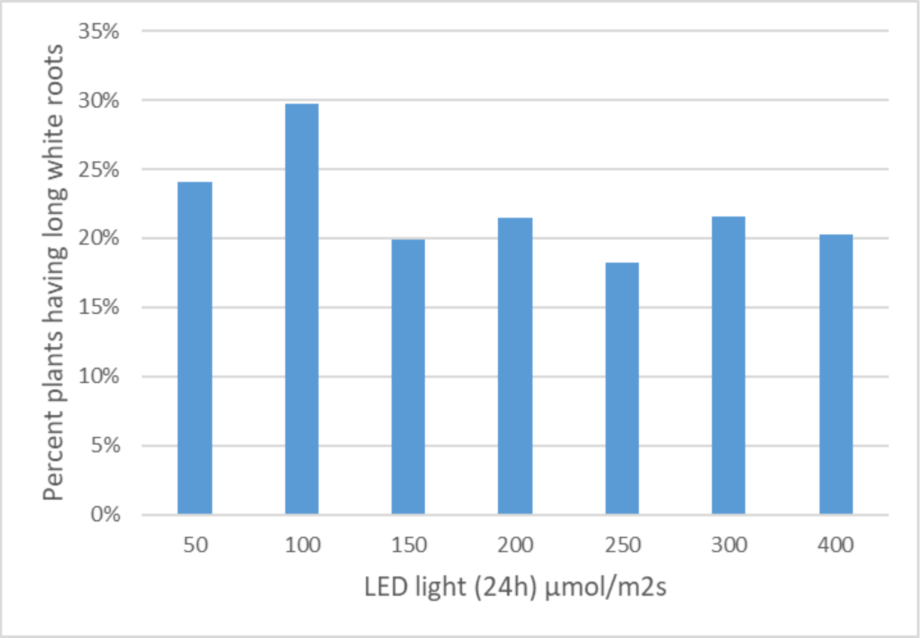

Figure S5. Percent plants having long white roots (score 2) as function of light intensity – average across all temperatures and clones.

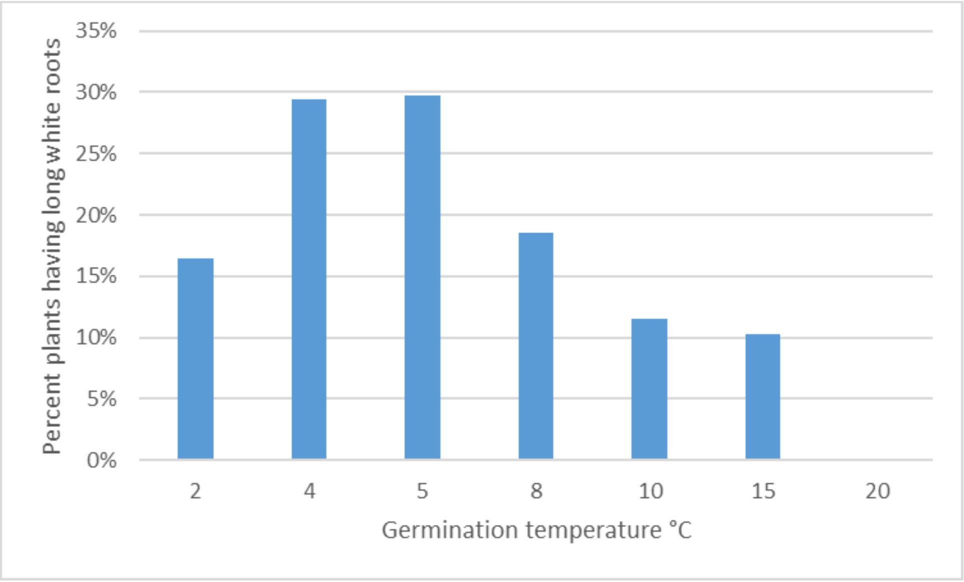

Figure S6. Percent plants having long white roots (score 2) as function of germination temperature – average across all clones.
