# Supplementary material for: Accumulated effects of factors determining plant development from somatic embryos of Abies nordmanniana and Abies bornmuelleriana
Source: Front Plant Sci. 2022 Oct 13;13:989484. doi: 10.3389/fpls.2022.989484 (PMC9608518; doi:10.3389/fpls.2022.989484)

Supplementary material.
Photos taken during germination at week 0, 3 and 8

Rooting in the dark - Clone : N1

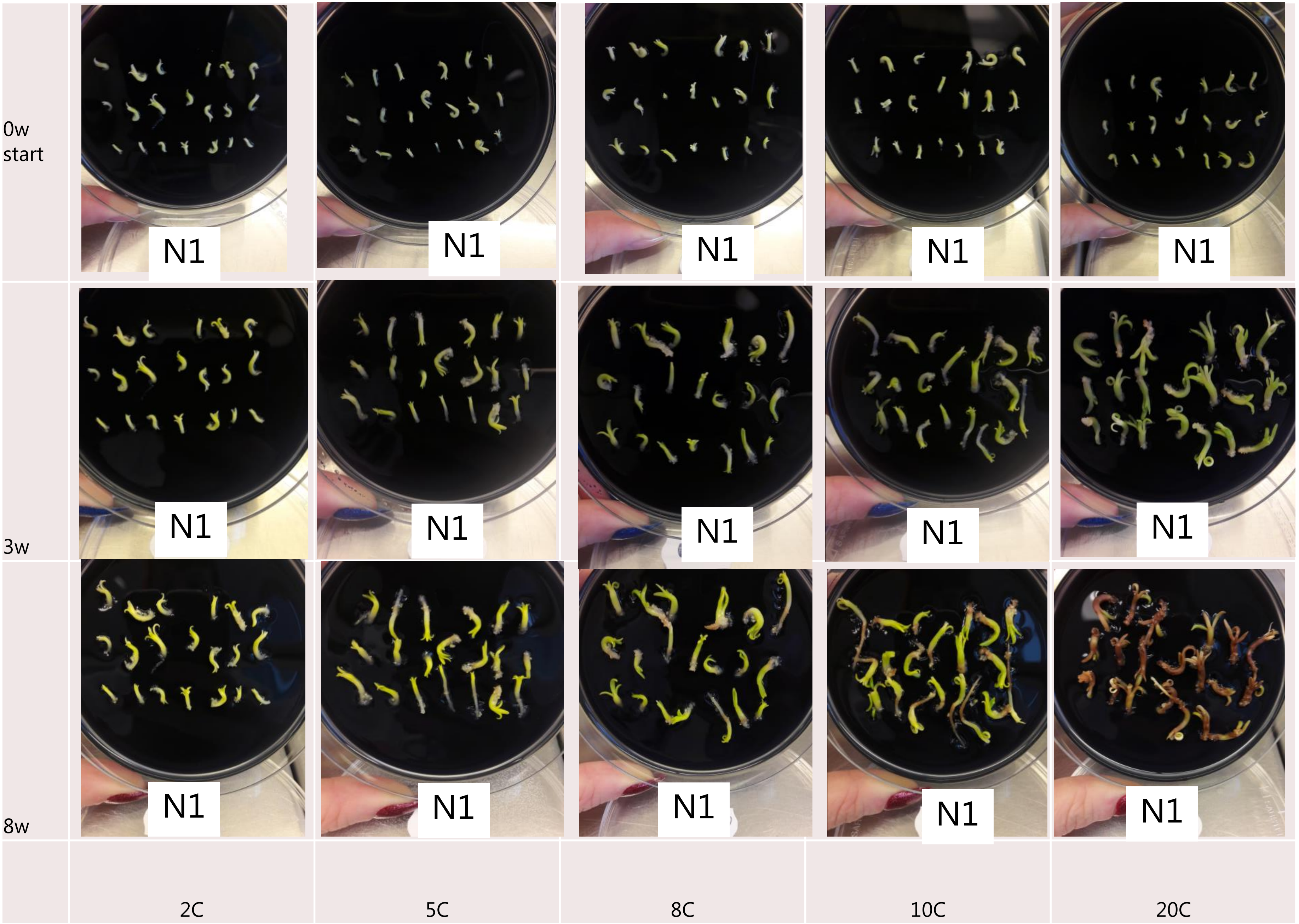

Rooting in the dark - Clone : N2

0w start

3w

8w

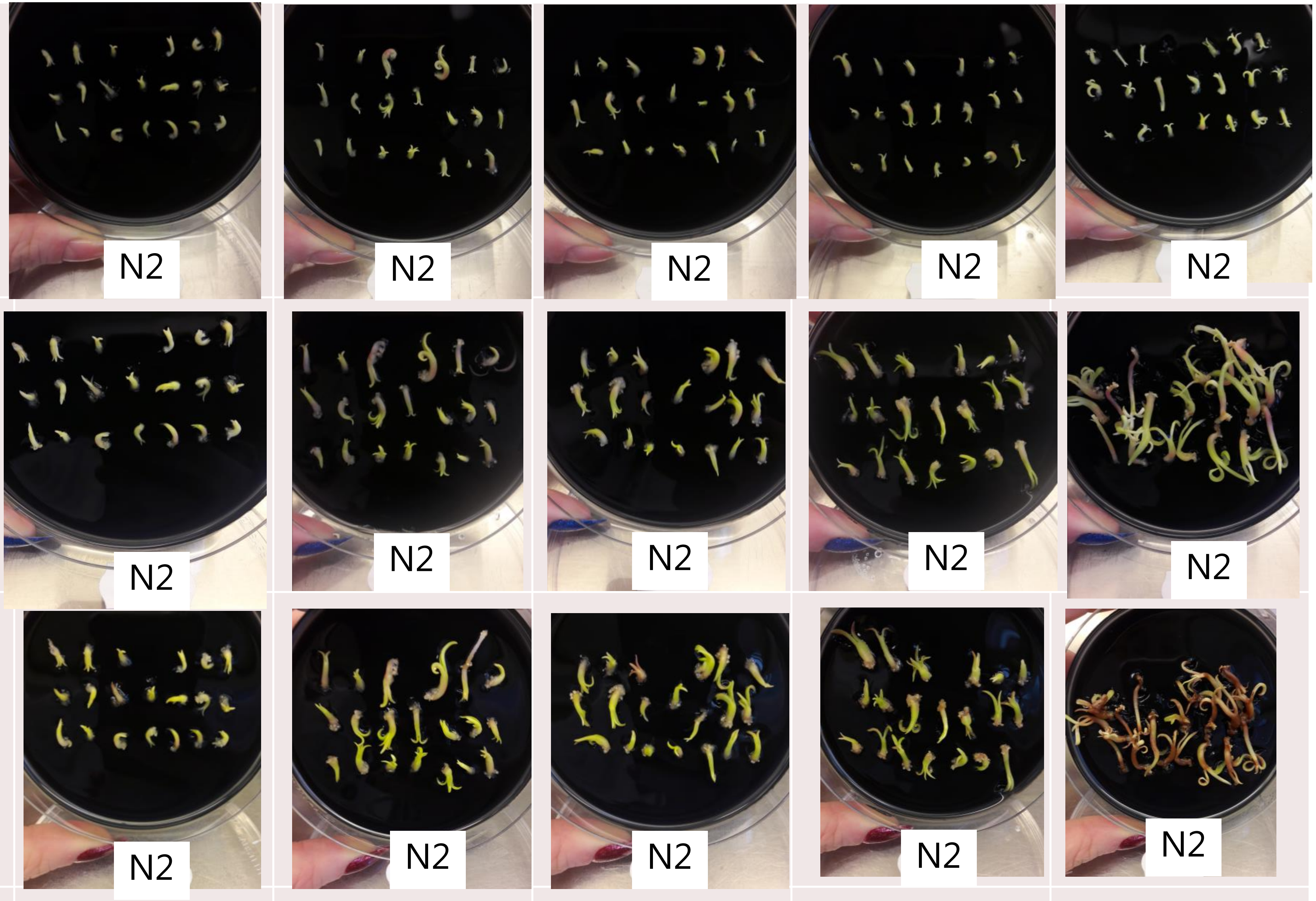

2C | 5C | 8C | 10C | 20C

Rooting in the dark - clone N3

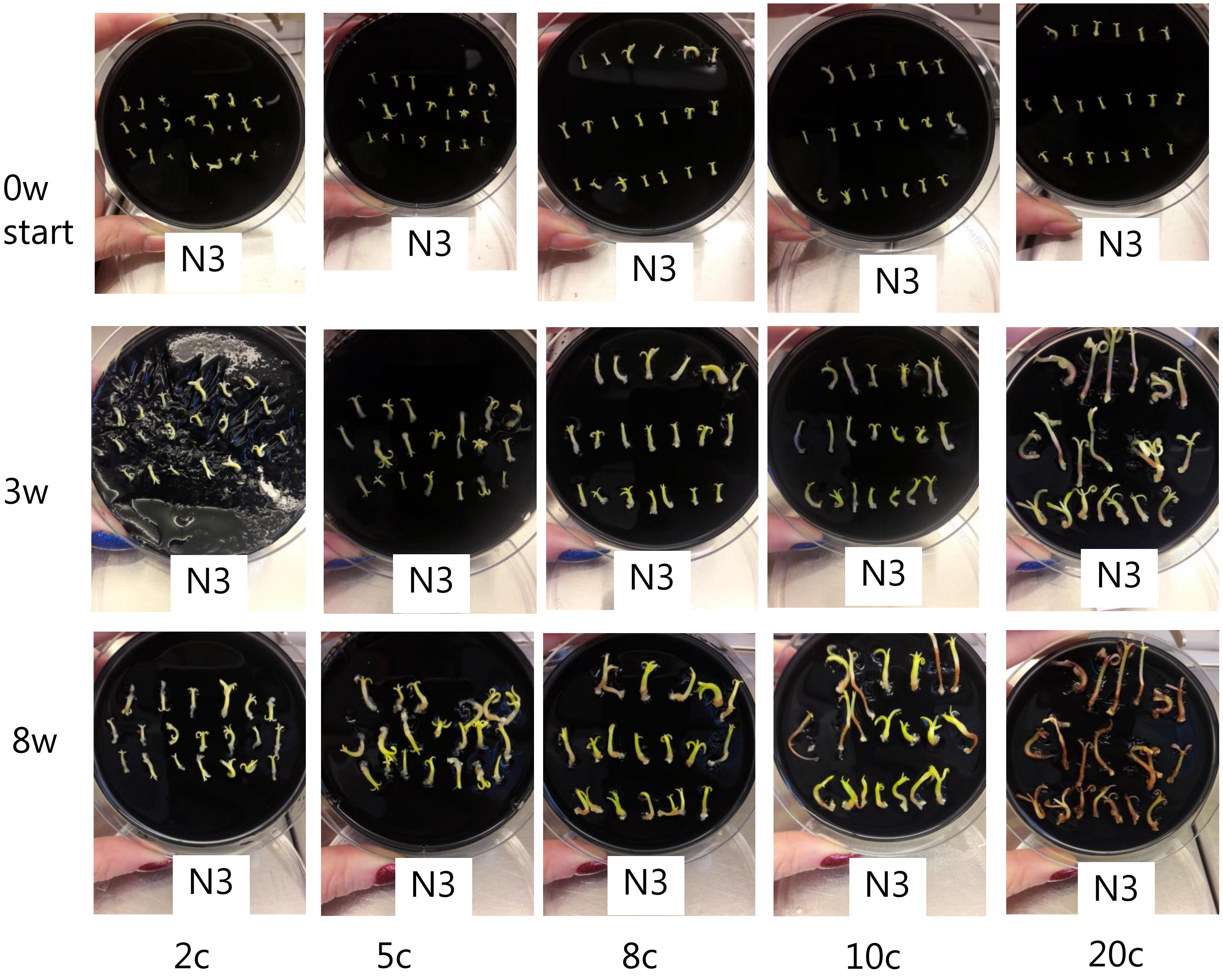

Rooting in the dark - Clone : N4

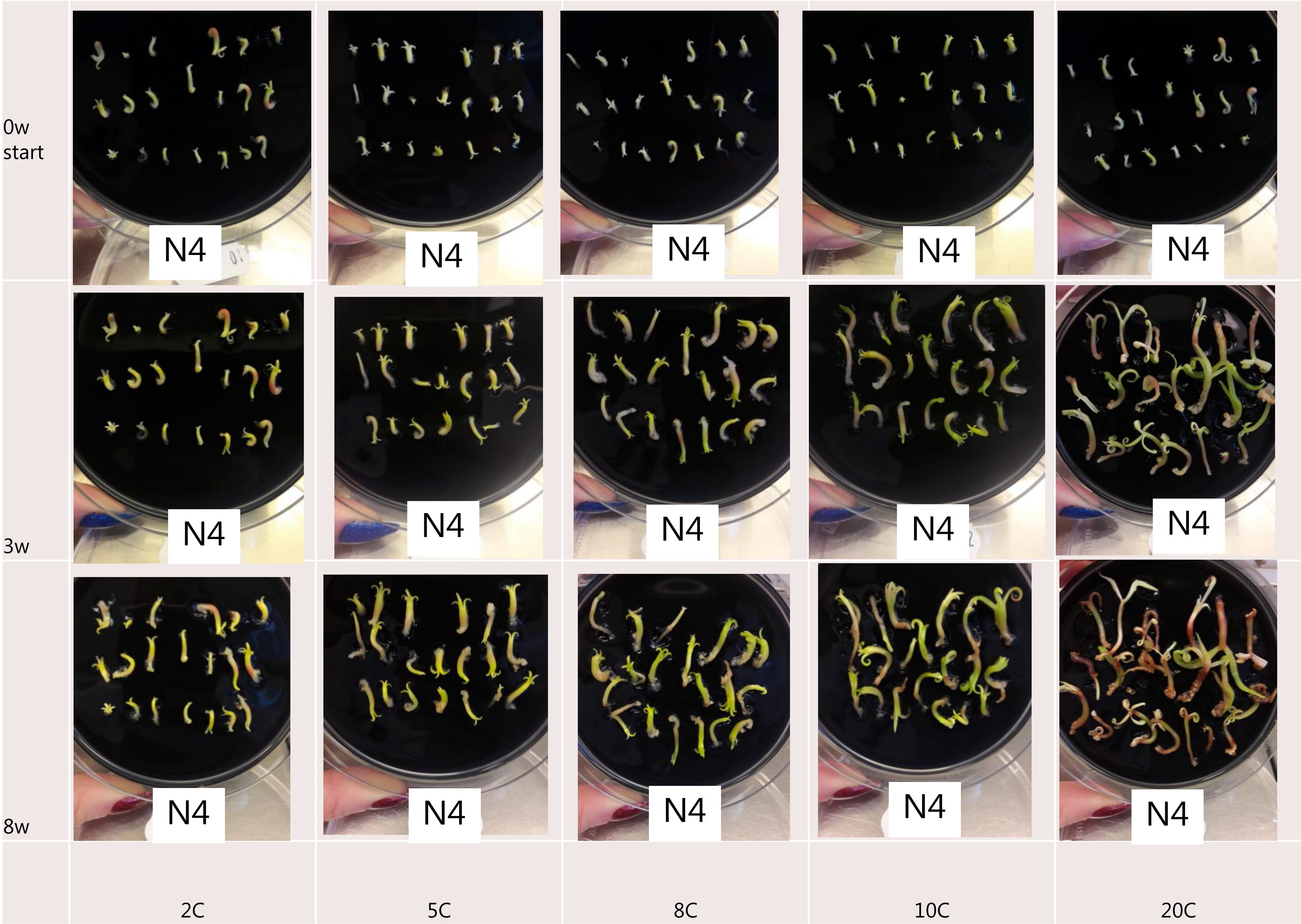

Rooted in the dark - Clone : N5

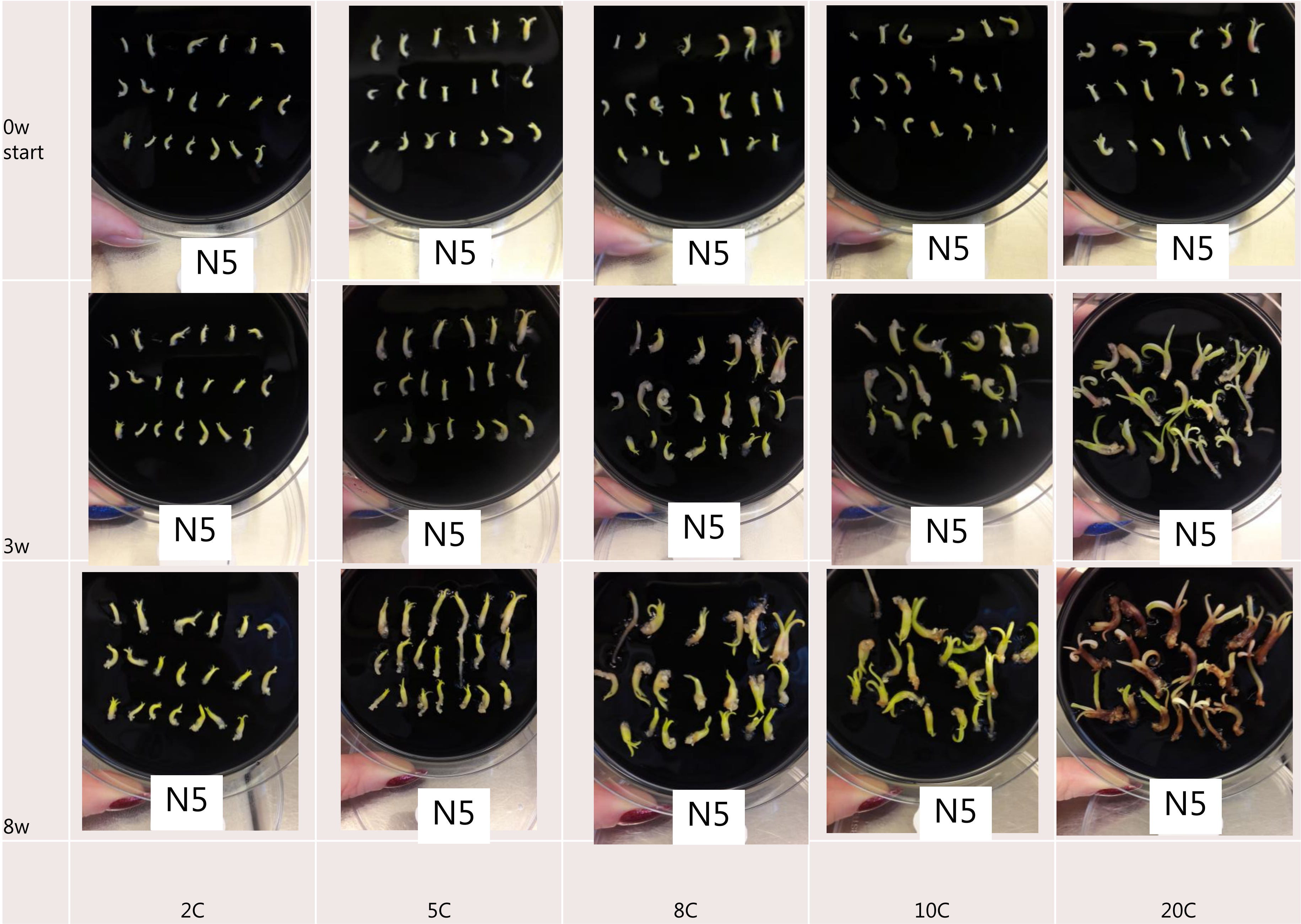

Rooting in the dark - Clone : N6

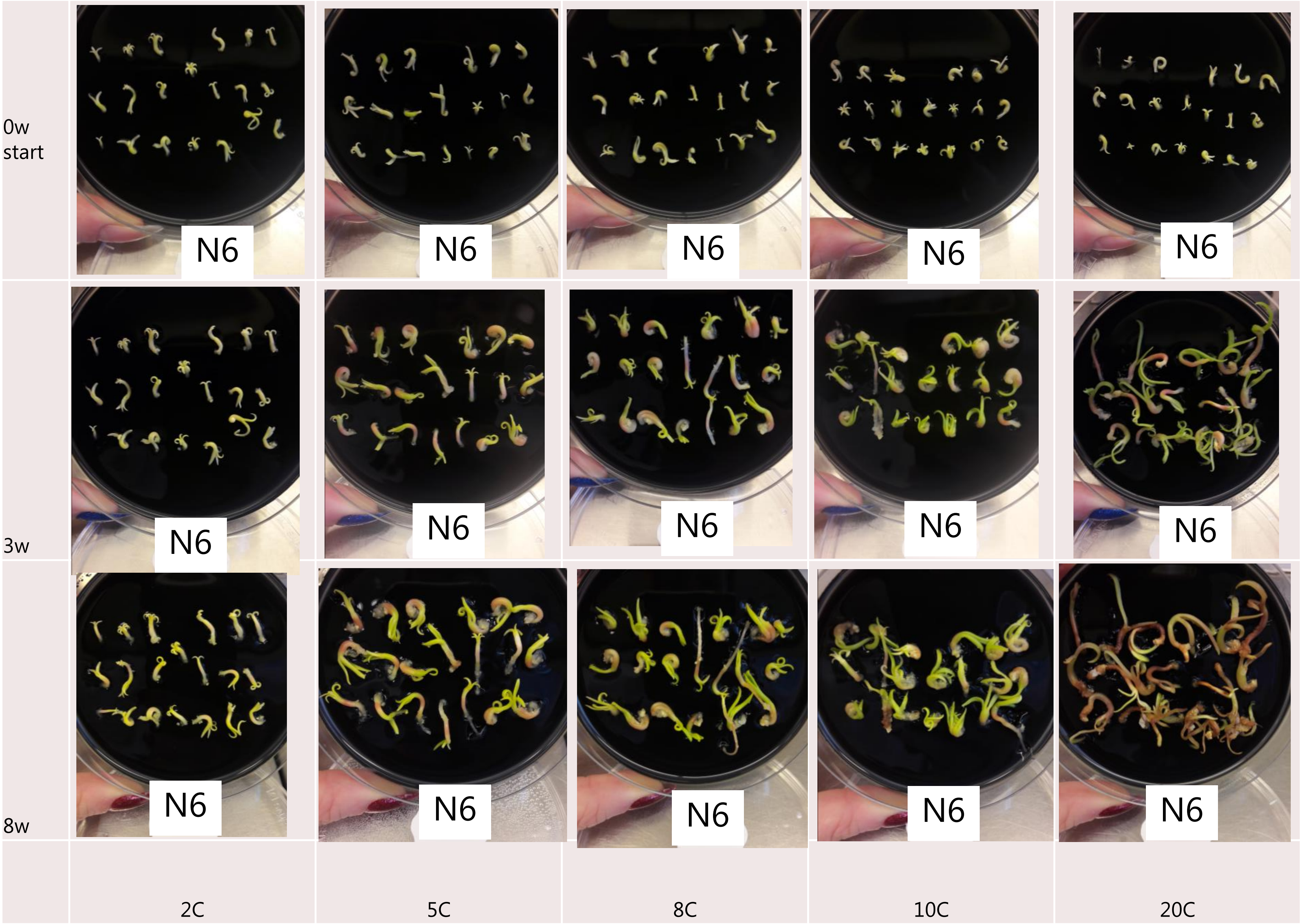

Rooted in the dark - Clone : N7

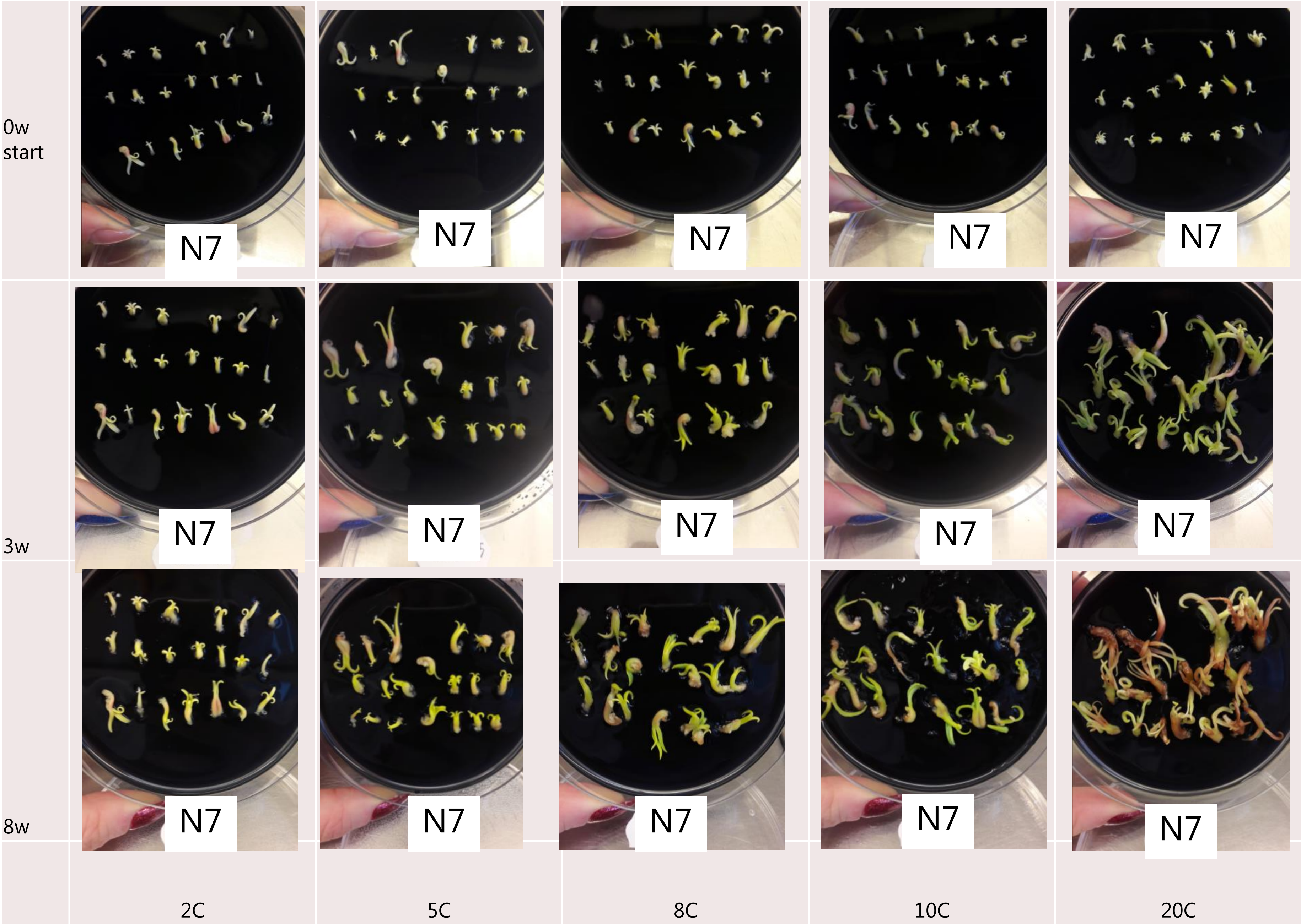

Rooted in the dark - Clone : T1

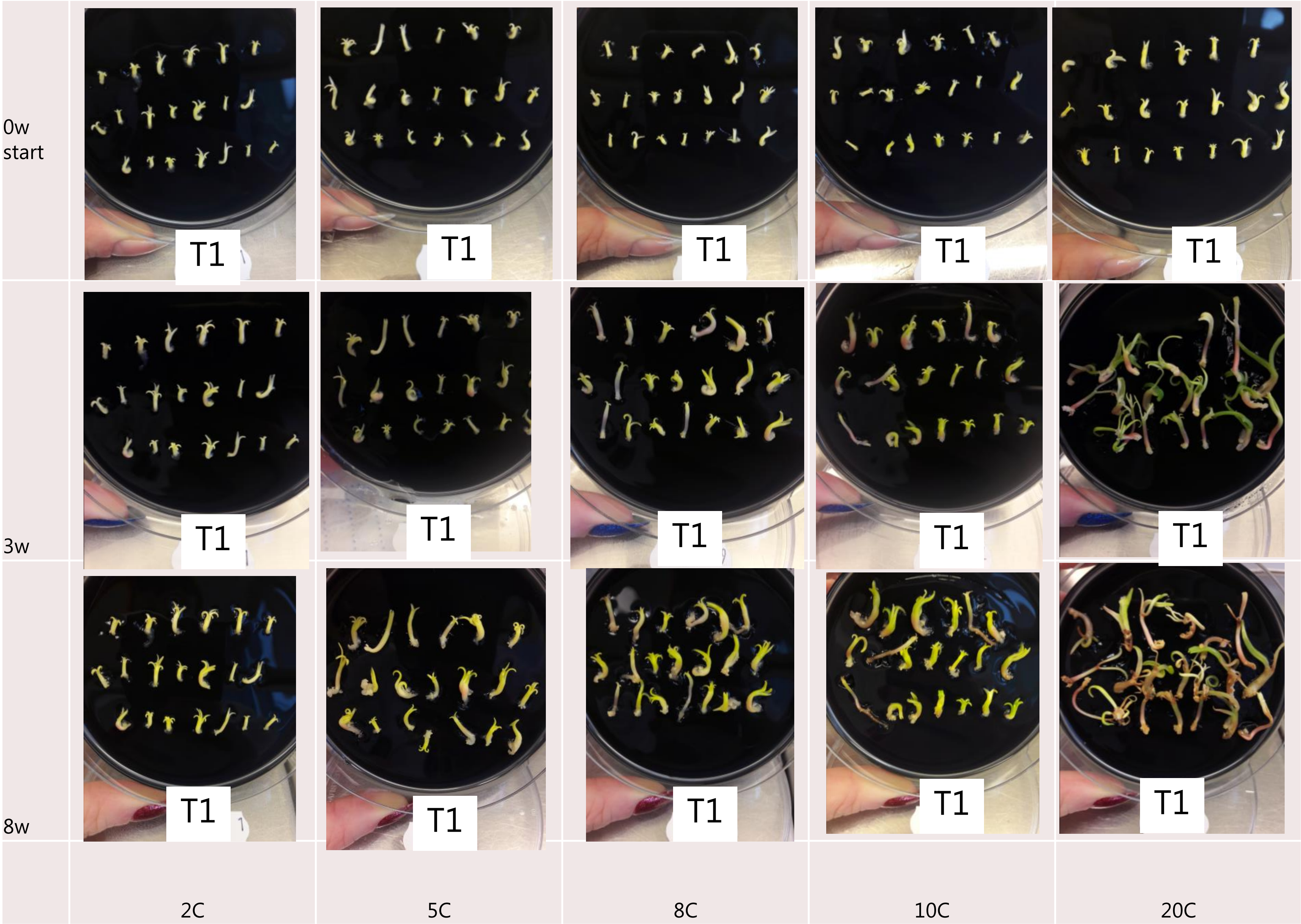

Rooted in the dark - Clone : T2

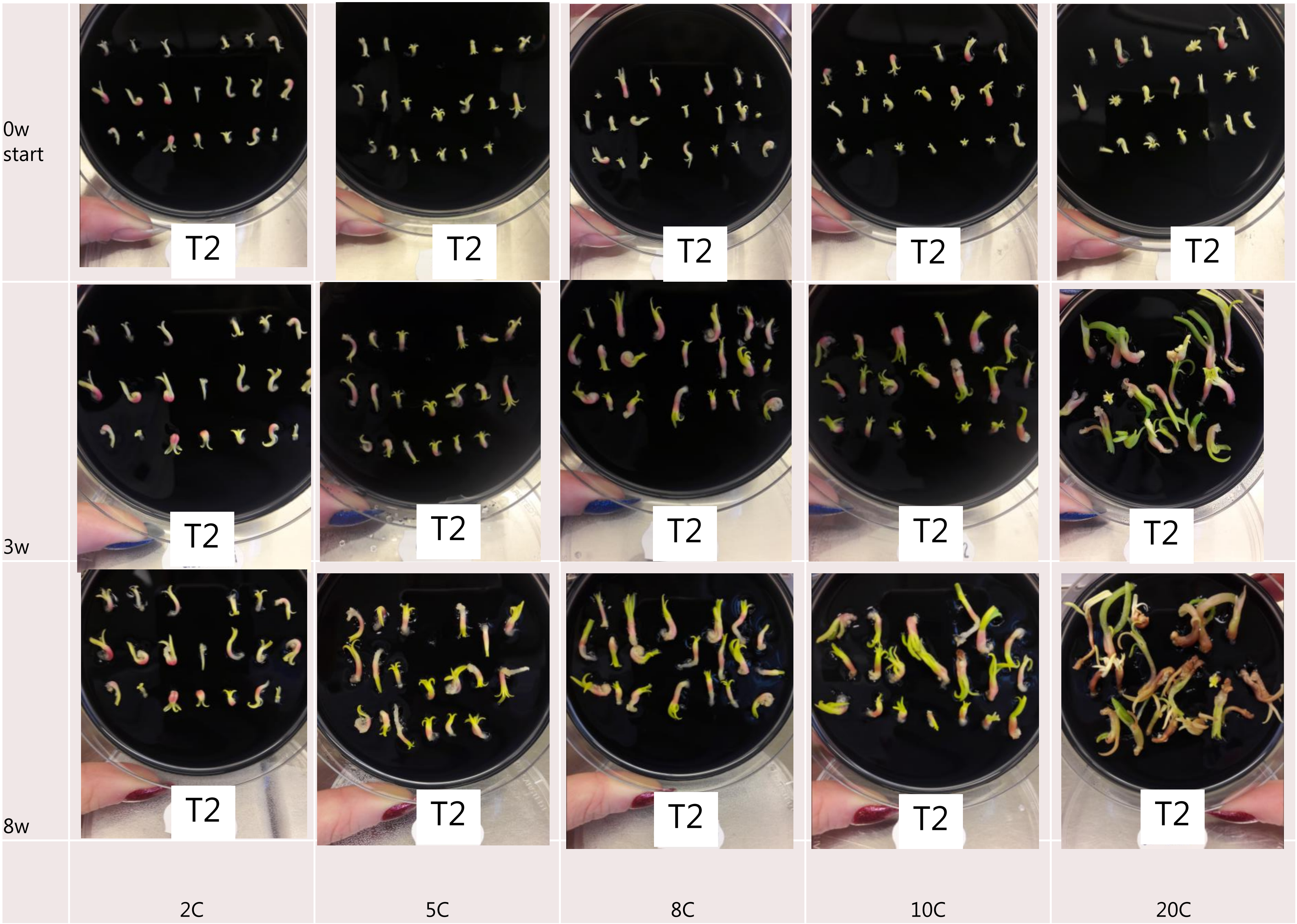

Supplement: Supplementary file 2 [file DataSheet_2.pdf]
